# HMGB proteins are required for sexual development in *Aspergillus nidulans*

**DOI:** 10.1371/journal.pone.0216094

**Published:** 2019-04-25

**Authors:** Eszter Bokor, Judit Ámon, Kabichandra Keisham, Zoltán Karácsony, Csaba Vágvölgyi, Zsuzsanna Hamari

**Affiliations:** University of Szeged, Faculty of Science and Informatics, Department of Microbiology, Szeged, Hungary; Woosuk University, REPUBLIC OF KOREA

## Abstract

*Aspergillus nidulans* has three high mobility group box (HMGB) proteins, HmbA, HmbB and HmbC that are chromatin-associated architectural proteins involved in DNA-related functions. By creating and studying deletion strains in both *veA*^*+*^ and *veA1* background, we have characterized the role of HmbA, HmbB and HmbC in sexual development. Expression of the mating-type MAT1-1 and MAT1-2 coding genes were found to be extremely down-regulated in all three mutants on day 4 of sexual development, which results in deficient ascospore production and/or ascospore viability in the mutants. In addition, we found that HmbA and HmbB play also a role in sensing of and response to environmental signals, while HmbC functionally interacts with VeA, a key regulator of the coordination of asexual and sexual development, as well as of secondary metabolism.

## Introduction

Beside linker histones (H1 and H5), B-type high mobility group box domain proteins (HMGB) (for nomenclature of HMG proteins see [1]) are also important architectural components of chromatin. These proteins can bind to linker DNA, and induce or repress gene expression. They are able to interact with both DNA and protein components of chromatin through their high mobility group box (HMG-box) domains (reviewed in [2–7]). The HMG-box domain (comprising three α-helices) folds up into an L-shape three dimensional form, and binds to the minor grove of DNA-helix, distorting the DNA’s backbone (reviewed in [4]). In HMGB proteins the amino acid that precedes the second α-helix of the HMG-box has non-polar characteristics, which endows the HMG-box with the ability to bind to the DNA with no or little sequence-specificity [3, 8]. HMGB proteins (e.g. human HMGB proteins, *Drosophila* DSP1, yeast HMO1 and HMO2) are generally composed of two or more copies of HMG-box domains, although single-copy HMG-box containing HMGB proteins also exist [4, 9, 10]. Three architectural HMGB proteins, namely HmbA (AN2885), HmbB (AN1267) and HmbC (AN10103), were identified in *Aspergillus nidulans* according to their diagnostic non-polar amino acids that precede the second α-helix in their canonical HMG-box [11]. Their closest characterized fungal homologues are Nhp6A/Bp, Abf2p and Hmo1p in *Saccharomyces cerevisiae* (homologues of HmbA, HmbB and HmbC, respectively) and mtHMG1 in *Podospora anserina* (homologue of HmbB) [9–15]. Of these three *A*. *nidulans* proteins, the physiological role of the dually localized mitochondrial/nuclear HmbB was previously characterized in details [11, 16]. Deletion of *hmbB* has pleiotropic effects (S1 Table). The *hmbBΔ* mutant is viable, but displays severe fitness loss due to the drastic reduction in the viability of conidiospores and ascospores (0.4% viable) [11]. Besides its role in spore viability, HmbB is also involved in diverse biological processes such as sugar metabolism during conidia germination, sterigmatocystin production, redox homeostasis or maintaining mitochondrial DNA copy number [11, 16]. The latter correlates with the mitochondrial localization of HmbB, whereas the other functions might be associated with the nuclear localization of the protein. This indicates that HmbB may play a role in the structure and function of nuclear chromatin [11]. This hypothesis is further supported by the altered transcription level of numerous nuclear genes in *hmbBΔ* [11, 16]. Regarding HmbA and HmbC, we have limited knowledge about their function in terms of their physiological roles and transcriptional regulation, however, based on the functions of their yeast homologues, it can be assumed that they modulate the expression of a wide range of genes [10, 14, 17, 18].

In *S*. *cerevisiae*, five HMGB proteins were identified, namely Nhp6Ap, Nhp6Bp, Hmo1p, Hmo2p, and Abf2p (physiological functions are summarized in S1 Table). The functionally redundant paralogue proteins, Nhp6Ap and Nhp6Bp (Nhp6A/Bp, homologues of HmbA), are composed of a single HMG-box domain, and act in the form of homodimers. Double deletion of *nhp6A* and *nhp6B* exerts a pleiotropic physiological effect. Specifically, the mutant shows morphological and cytoskeletal defects, such as sensitivity to starvation and growth defect at high temperature settings, which can be suppressed by adding an osmotic stabilizer in the medium [19]. At the molecular level, the functions of Nhp6A/Bp proteins are similar to that of mammalian HMGB1. Specifically, they regulate the transcription of Pol II-dependent genes at the genome-scale level through different mechanisms. They modulate the interaction of TATA-binding protein (TBP) to the promoter sites and the subsequent formation of the TBP/TFIIA/DNA complexes [20–22]. They also interact with the yeast FACT complex, which ensures transcription elongation by RNA Pol II through nucleosomal templates by removing histone H2A-H2B dimers [23]. In addition, they also interact with transcription activators or repressors [24]. The Nhp6A/Bp proteins are also important mediators of the expression of RNA Pol III-transcribed genes [25].

Prominent examples of HMGB proteins having two copies of HMG-box domains are Hmo1p, Hmo2p and Abf2p. Deletion of the Hmo1p (homologue of HmbC) coding gene results in a severe growth defect (with extremely reduced colony size), reduced plasmid stability, hypersensitivity to micrococcal nuclease, and a decrease in transcription executed by RNA Pol I and II [9]. At the molecular level, Hmo1p promotes the transcription of rRNA genes [10], as well as interacts with Fhl1p (regulator of ribosomal protein genes) [26], TBP and TFIID [17]. Deletion of the Hmo2p coding gene results in the weakening of the DNA double-strand break repair mechanisms mediated by INO80 complex and γ-H2A.X [27]. At the molecular level, Hmo2p protects broken DNA from exonucleolytic cleavage by recognizing and binding to DNA ends [28], and recruits the INO80 complex to the damage-induced phosphorylated γ-H2A.X [29]. Deletion of the Abf2p (homologue of HmbB) coding gene has pleiotropic effects including the loss of compaction of the mitochondrial nucleoid, the decrease of mitochondrial recombination frequency and the decrease of mitochondrial genome stability on fermentable carbon sources. These effects are the consequences of DNA bending and wrapping, as well as the promotion and stabilization of Holliday-junction recombination intermediates [30–32].

*P*. *anserina* has five HMGB proteins (PaHMG2, PaHMG3, PaHMG4, PaHMG6 and mtHMG1) (physiological functions are summarized in S1 Table) and two putative HMGB proteins (PaHMG7 and PaHMG9) [33]. Of these seven proteins, mtHMG1 localizes to mitochondria (similarly to yeast Abf2p and HmbB of *A*. *nidulans*) and is essential for the maintenance of mitochondrial genome. In the presence of an extra copy of this gene, the ‘premature death’ phenotype originating from mitochondrial DNA rearrangements in an *AS1-4* context is partially suppressed [12]. The *mthmg1* deletion strain displays altered germination, growth and fertility, as well as reduced life span in *AS1*^*+*^ context [12]. All but one (PaHMG2) of the *P*. *anserina* HMGB type proteins were shown to be implicated in certain aspects of sexual development [33]. Two of them, PaHMG6 and mtHMG1 (orthologues of *A*. *nidulans* HmbA and HmbB, respectively), govern the expression of transcription factors required for sexual development, such as the α-box mating-type FMR1, the HMG-box mating-type transcription factor FPR1 and mating-type HMG-box domain containing transcription factors PaHMG5, PaHMG8 and PaHMG9 [33].

The sexual development of *A*. *nidulans* (homothallic, self-fertile) is regulated by various environmental factors (light, oxygen level, nitrogen source, pH), and requires the contribution of regulatory proteins, such as the velvet family protein VeA and mating-type factors MAT1-1 and MAT1-2 [34, 35]. Deletion of the *veA* gene results in a complete loss of the ability to undergo sexual development, while the *veA1* mutation (start codon ATG mutated to ATT) results in an N-terminal truncated protein devoid of nuclear localization signal and evokes a light-independent functioning for the protein [36]. The *veA1* mutant strains produce conidia abundantly even in dark conditions, produce few aerial hyphae and form fruiting bodies regardless of light conditions, although at a reduced level compared to that of *veA*^*+*^ strains [37].

During the sexual development of *A*. *nidulans*, thick-walled Hülle cells are formed, which surround an aggregated hyphal mass (primordium) composed of differentiated cells of the pericarp (outside) and ascogenous hyphae (inside). Subsequently, a tiny immature cleistothecium (μ-cleistothecium) develops from the primordium, which is composed of non- or lightly-pigmented pericarp cells and ascogenous hyphae with ascus mother cells. At later stages of the development, immature asci are formed with 8 non- or lightly-pigmented ascospores [38]. During maturation, the immature cleistothecium transforms to mature cleistothecium by the accumulation of a dark pigment in the wall cells and binucleate ascospores having an oval shape [38]. In our current research we have investigated HmbA, HmbB and HmbC of *A*. *nidulans*, orthologues of PaHMG6, mtHMG1 and PaHMG4 of *P*. *anserina* [33]. By studying the sexual structures of *hmbA*, *hmbB* and *hmbC* deletion mutants and analyzing gene expression of mating-type genes, we have revealed that the HMGB proteins play an essential role in ascospore production and viability, possibly via their modulation of the expression of mating-type genes that influence the late-stage sexual development of *A*. *nidulans*.

## Materials and methods

### Strains, media and growth conditions

The *A*. *nidulans* strains used in this study are listed in S2 Table. Standard genetic markers, complete medium (CM) and minimal medium (MM) are described at the following URL: http://www.fgsc.net/Aspergillus/gene_list/. Media were supplemented with vitamins (www.fgsc.net) according to the requirements of each auxotrophic strain. Agar for minimal media was obtained from BD/Difco. For total DNA extraction, 10^8^ conidiospores were inoculated to 100 ml liquid MM and incubated at 37°C for 14–16 h with 180 rpm shaking. Sexually developing mycelia for RNA extraction were prepared according to Zheng *et al*. [39]. 10^8^ conidiospores were inoculated to 300 ml MM and incubated at 37°C for 24 h with 180 rpm shaking. For the induction of sexual development, the mycelia from the 24 h cultures were transferred to solid MM plates and sealed tightly with scotch tape. The plates were incubated in complete darkness at 37°C for 48 h and 96 h prior to RNA extraction.

### Construction of deletion and complementation strains

Deletion of *hmbB* had been reported previously [11]. Deletions of *hmbA* and *hmbC* were obtained by the transformation of tripartite gene-substitution cassettes constructed by the double-joint PCR method [40] as described previously [11]. The cassettes were composed of "A", "B" and "C" components. The flanking "A" and "C" components contained a 2–3 kb fragments of genomic region upstream and downstream to the target locus, respectively, while the middle "B" components contained the selection marker gene *riboB*^*+*^ (in case of *hmbA* deletion) or a *pabaA*^*+*^ (in case of *hmbC* deletion). Used primers are listed in S3 Table. The "A", "B" and "C" components of the *hmbA* substitution cassette were amplified with the "hmbA up frw"–"hmbA up rev", "hmbA ribo chim frw"–hmbA ribo chim rev" and "hmbA down frw"–"hmbA down rev" primer pairs, respectively. The 3,428 bp, 2,208 bp and 3,058 bp long "A", "B" and "C" components were assembled to a 7,827 bp long substitution cassette using "hmbA up nest frw" and "hmbA down nest rev" primers. The substitution cassette was transformed into HZS.120 and 30 riboflavin prototrophic strains were selected and pre-screened for the deletion by PCR using "HmbA frw" and "HmbA rev" primers. Single copy integration mutants were selected on the basis of Southern blot analysis using the DIG-labeled "C" component as DNA probe on XbaI digested total DNA (S1 Fig). One single copy integration mutant (HZS.205) was used in genetic crosses to obtain *veA*^*+*^ and v*eA1 hmbAΔ* strains (HZS.521 and HZS.239, respectively) for further studies. Reconstitution of *hmbA* deletion was obtained by cloning the *hmbA* product (amplified by "hmbA prom NotI frw" and "hmbA term NheI rev" primers) into the NheI/NotI sites of pAN-HZS-1 vector [11] (see more in S1 Materials and methods) and transforming the developed pAN-HZS-9 vector into the recipient strains HZS.320 (*hmbAΔ*, *veA1*) and HZS.655 (*hmbAΔ*, *veA*^*+*^). Out of 50 transformants of HZS.320 and 19 transformants of HZS.655, 4 carried single copy integration (cross-checked by qPCR with "hmbA ReTi frw" and "hmbA ReTi rev" primers, respectively). The single copy integration strains HZS.621 (reconstituted *hmbAΔ*, *veA1*) and HZS.678 (reconstituted *hmbAΔ*, *veA*^*+*^) were used in further studies.

The "A", "B" and "C" components of the *hmbC* substitution cassette were amplified with the "hmbC up frw"–"hmbC up rev", "hmbC paba chim frw"–"hmbC paba chim rev" and "hmbC down frw"–"hmbC down rev" primer pairs, respectively. The 2,634 bp, 3,846 bp and 2,320 bp long "A", "B" and "C" components were assembled to a 8,069 bp long substitution cassette using "hmbC up nest frw" and "hmbC down nest rev" primers. The substitution cassette was transformed into HZS.314 and 30 p-amino-benzoic acid prototroph strains were selected and pre-screened for the deletion by PCR using "HmbC frw" and "HmbC rev" primers. Single copy integration mutants were selected on the basis of Southern blot analysis using the DIG-labeled "C" component as DNA probe on EcoRV digested total DNA (S2 Fig). One single copy integration mutant (HZS.338) was used in genetic crosses to obtain prototrophic *veA*^*+*^ and v*eA1 hmbCΔ* strains for the further studies. Reconstitution of *hmbC* deletion was obtained by cloning the *hmbC* PCR product (obtained by using "hmbC NcoI frw" and "hmbC BamHI rev" primers) into the NcoI/BamHI sites of pAN-HZS-1 vector [11] (see more in S1 Materials and methods) and transforming the obtained pAN-HZS-10 into the recipient strains HZS.338 (*hmbCΔ*, *veA1*) and HZS.658 (*hmbCΔ*, *veA*^*+*^). Out of 16 transformants of HZS.338 and 13 transformants of HZS.658, 2 and 3 carried single copy integration (checked by qPCR with "hmbC ReTi frw" and "hmbC ReTi rev" primers), respectively. The single copy integration strains HZS.676 (reconstituted *hmbCΔ*, *veA1*) and HZS.679 (reconstituted *hmbCΔ*, *veA*^*+*^) were used in further studies.

Reconstitution of *hmbBΔ* was obtained by cloning the *hmbB* PCR product (obtained by using "hmbB NcoI frw" and "hmbB BamHI rev" primers) into the NcoI/BamHI sites of pAN-HZS-1 vector [11] (see more in S1 Materials and methods) and transforming the obtained pAN-HZS-11 vector into the recipient strains HZS.318 (*hmbBΔ*, *veA1*) and HZS.653 (*hmbBΔ*, *veA*^*+*^). Out of 2 transformants of HZS.318 and 23 transformants of HZS.653, one and 3 carried single copy integration (checked by qPCR with "hmbB ReTi frw" and "hmbB ReTi rev" primers), respectively. The single copy integration strains HZS.677 (reconstituted *hmbBΔ*, *veA1*) and HZS.680 (reconstituted *hmbBΔ*, *veA*^*+*^) were used in further studies.

### DNA manipulations

DNA was prepared from *A*. *nidulans* as described by Specht et al. [41], and hybond-N membranes (Amersham/GE Healthcare) were used for Southern blots [42]. Southern hybridizations were done by DIG DNA Labeling and Detection Kit (Roche) according to the manufacturer's instructions. Transformations of *A*. *nidulans* protoplasts were performed as described by Antal et al. [43]. The protoplasts were prepared from mycelia grown over cellophane [44, 45] using a 1% solution of Glucanex (Novozymes, Switzerland) in 0.7 M KCl. Transformation of 5x10^7^ protoplasts was carried out with 100–500 ng of fusion PCR products or plasmid vectors.

### Genetic crosses

Heterozygotic crosses were done according to Pontecorvo [38]. The *veA* allele of each *veA*^*+*^ progeny selected for further work was checked by using the "veA DM frw"–"veA DM rev" and "veA frw"–"veA rev" primer pairs [46] and also sequenced with the "veA seq frw" primer. Homozygotic crosses were always self-crosses (selfing or inbreeding) made by sealing (in the case of *hmbBΔ* selfing the plates were not sealed for obtaining normal-sized fruiting bodies) of 2-day-old CM agar cultures with scotch tape. Inbreeding plates were inoculated in replicates and kept in complete darkness at 37°C for different time periods. Each day following the fourth day of incubation one of the replicate plates was opened and the sexual structures were studied under stereo- and light microscopes.

### Microscopy

Cleistothecia were taken from colonies by a needle and the samples were mounted on slides directly or after their cleaning (by rolling them on the surface of an agar plate to eliminate mycelia, conidiospores and Hülle cells) using 1x PBS buffer. After crushing the samples between the slide and cover slip, the sexual structures were documented. DIC images were taken by using Olympus BX51 microscope.

### Quantitative PCRs and data analysis

To assess the copy number of integration events in the transformant strains, total DNAs were extracted [41] and used in quantitative real-time PCR (qPCR) using *hmbA*, *hmbB*, *hmbC* and reference gene *actA* (AN6542) specific primer pairs (S3 Table). For reverse transcription-qPCR (RT-qPCR), total RNA was isolated from sexually developing mycelia [39] by using RNeasy Plus Mini Kit (Qiagen) or TRIsure reagent (Bioline) with RNase-Free DNase Set (Qiagen) according to the manufacturer's instructions (for further details, see S1 Materials and methods). cDNA synthesis was carried out on 1 μg RNAs with a mixture of oligo-dT and random primers using the RevertAid First Strand cDNA Synthesis Kit (Fermentas). Quantitative PCRs were performed in a CFX96 Real Time PCR System (BioRad) with SYBR Green/Fluorescein qPCR Master Mix (Fermentas) reaction mixture and specific primers listed in S3 Table. Five reference genes (*actA*, histone H3 *hhtA*/AN0733, beta-tubulin *tubC*/AN6838, Glyceraldehyde-3-phosphate dehydrogenase *gpdA*/AN8041 and Elongation factor 3 *eEF-3*/AN6700) were tested by using geNorm (https://genorm.cmgg.be). The *gpdA* and *tubC* genes were selected to obtain a ‘gene expression normalization factor’ (for further details, see S1 Materials and methods). Transcript levels were estimated according to the relative standard curve method [47], using three technical replicates of three biological samples.

### Determination of the size of cleistothecia and germination ability of ascospores

In order to determine the size of the cleistothecia, a mass of cleistothecia was taken out from one spot of the colonies. Ten individual cleistothecia that represented the frequency of the observed sizes were purified and documented in the presence of a size ruler by a camera. The average sizes of the cleistothecia were determined by conducting image analysis on individual cleistothecia using Adobe Photoshop. The germination ability of the ascospores was determined by counting colony-forming units after inoculating 100 ascospores on solid medium. Dilutions of 10^5^−10^7^ ascospores were plated onto MM agar plates and incubated at 37°C for 2–4 days. The numbers of colonies were counted and normalized to 100 ascospores. All experiments were performed on at least ten biological replicates in three technical replicates.

### Statistical analysis

All statistical analysis was performed using GraphPad Prism version 5.02 for OSX (GraphPad Software, San Diego, CA). The significant differences between sets of data were determined by one-way ANOVA test, two-way ANOVA or Mann-Whitney *U*-test according to the data.

## Results

### Generation of deletion strains and analysis of the vegetative development

In order to study the role of the three HMGB proteins of *A*. *nidulans* in sexual development, we generated *hmbAΔ*, *hmbBΔ* and *hmbCΔ* deletion strains both with a *veA*^*+*^ and a *veA1* genetic background. In case of *hmbB*, we used a previously generated mutant strain, the *hmbBΔ veA1* (HZS.212) [11]. By performing genetic crossing, we obtained prototrophic *hmbBΔ veA1* strain (HZS.280) and *hmbBΔ veA*^*+*^ strain (HZS.495). The *hmbA* and *hmbC* deletion mutants were developed in a *veA1* genetic background (HZS.205 and HZS.338, respectively) via the transformation of gene-substitution cassettes constructed by the Double-Joint PCR method [40] into the appropriate recipient strains (HZS.120 and HZS.314, respectively) (for details see Materials and methods and S1 and S2 Figs). We developed the prototrophic or auxotrophic *hmbAΔ* and *hmbCΔ* strains with *veA1* (HZS.239 and HZS.338) and *veA*^*+*^ genetic backgrounds (HZS.521 and HZS.531) by performing genetic crosses. *In trans* expression of *P*_*hmbA*_*-hmbA*, *P*_*gpdA*_*-hmbB* and *P*_*gpdA*_*-hmbC* in the corresponding *hmbA*, *hmbB* and *hmbC* deletion strains (for details see the Materials and methods section and S1 Materials and methods) mitigated but did not completely abolish the deletion phenotype with respect to colony macromorphology and ascospore production/viability (see below). The reason for this partial complementation may be the *in trans* expression of the constructs and/or usage of the constitutive *gpdA* promoter (P_*gpdA*_) in the case of complementation of *hmbB* and *hmbC* deletion.

**Fig 1:**
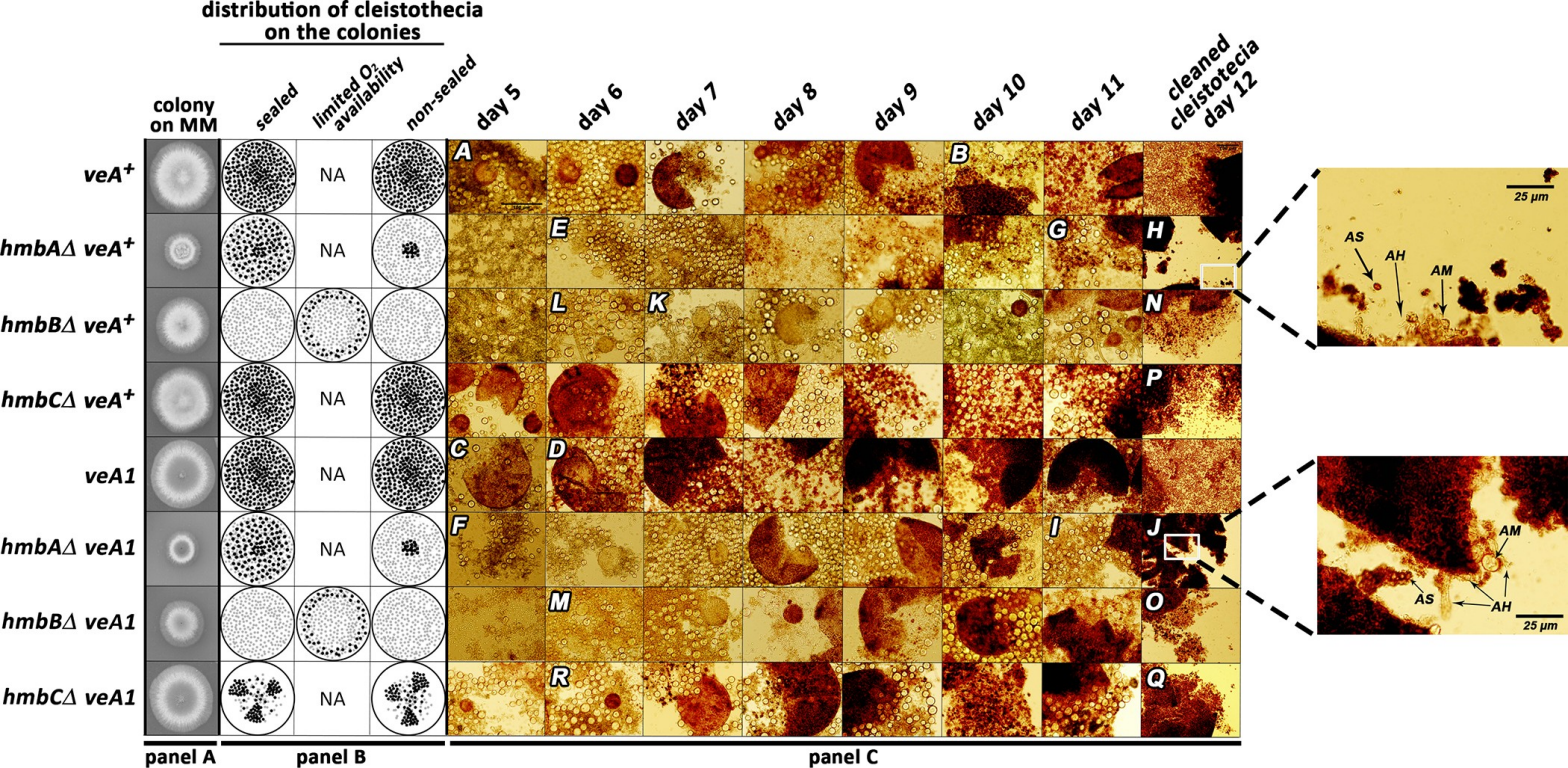
Documentation of sexual structures formed during the course of sexual development in control and *hmbA*, *hmbB* and *hmbC* deletion strains. Panel A: Growth ability of *veA*^*+*^ and *veA1* controls and the *hmbAΔ*, *hmbBΔ* and *hmbCΔ* strains in both *veA*^*+*^ and *veA1* background. The strains were incubated on CM for 2 days at 37°C prior documentation. Strains used: *veA*^*+*^ control (HZS.450), *veA1* control (HZS.145), *hmbAΔ veA*^*+*^ (HZS.521), *hmbAΔ veA1* (HZS.239), *hmbBΔ veA*^*+*^ (HZS.495), *hmbBΔ veA1* (HZS.280), *hmbCΔ veA*^*+*^ (HZS.531), *hmbCΔ veA1* (HZS.338). The complete genotypes are listed in S2 Table. Panel B: Schematic representation of oxygen regulated distribution pattern of cleistothecia in control and *hmbAΔ*, *hmbBΔ* and *hmbCΔ* strains. “Sealed” and “non-sealed” refer to conditions where plates are sealed with scotch tape or kept without sealing, respectively. “Limited oxygen availability” refers to the non-sealed condition with the applied medium almost entirely filling up the Petri dish. Small-sized grey dots mark micro-sized cleistothecia, black dots indicate normal-sized cleistothecia. Panel C: Time course of sexual structure formation in control and *hmbA*, *hmbB* and *hmbC* deletion strains. Selfing was done on CM at 37°C in complete darkness. In the case of *hmbBΔ* strains (both *veA*^*+*^ and *veA1*) the plates remained non-sealed and the medium almost entirely filled up the Petri dish. The rest of the selfing plates were sealed by scotch tape. Samples from the areas showing sexual development were taken each day from day 5 until day 11, and were studied without purification by using microscope. Cleistothecia collected from day 12 were purified on a sterile agar plate prior to mounting. Letters A-R correspond to images with landmark structures discussed in the Results section. Images A, E, F, K, L, M and R show primordia, image C shows μ-cleistothecium, images B, D, G, H, I, J, N, O, P and Q show cleistothecia with mature ascospores. These sexual structures can also be observed in the non-marked images. Images were taken by an Olympus BX51 microscope. The 100 μm scale bar shown on image A refers to all images except those in the day 12 column and magnifications of selected area on images I and K. The strains used in the experiment are the same as those listed in Panel A.

Macromorphological analysis of the mutant strains revealed that the *hmbCΔ* strain grown on MM or CM (Materials and methods) was not different from the *hmbC*^*+*^ control (Fig 1 and S3 Fig). The *hmbAΔ* strains formed smaller colonies compared to those of the *hmbA*^*+*^ controls, due to slower growth rate (detailed description of the *hmbAΔ* phenotype will be described elsewhere) (Fig 1 and S3 Fig). The *hmbBΔ* strains also formed smaller colonies compared to the controls, explained by delayed conidiospore germination rather than by a slower growth rate as it was described earlier [11] (Fig 1 and S3 Fig).

### Time course of sexual development and intracolonial distribution of cleistothecia

The sexual development of *veA*^*+*^ and *veA1* control strains, along with that of the *hmbAΔ*, *hmbBΔ* and *hmbCΔ* strains with both *veA*^*+*^ and *veA1* background were documented on a daily basis from day 5 to day 12 of incubation on selfing cultures (Fig 1) (for details see Materials and methods). All the deletion mutants were able to produce Hülle cells and cleistothecia with various severities of defects in ascospore formation and ascospore viability. This phenomenon was accompanied by the appearance of a reddish granular amorphous (RGA) material, which resembles to that observed in the barren cleistothecia of MAT1-1 and MAT1-2 protein coding gene deletion strains [35]. The comparative study of the time course of sexual development, intracolonial distribution, size and ascospore content of the cleistothecia revealed the role of HmbA, HmbB and HmbC proteins in sensing of and response to environmental factors, the production and viability of ascospores and their possible functional interaction with VeA functions. The observed phenotypes of the mutants are summarized in Table 1.

**Table 1:** Summary of the phenotypic analysis of *veA*^*+*^ control and deletion strains.

strain	time course of sexual development	median ascospore number per cleistothecium (n = 10)	viability of ascospores (n = 10)	RGA material	size of cleitothecia (n = 10)	distribution of cleistothecia
**wt** **(*veA***^***+***^**)**	wild-type	3.7×10^5^ ±1.9×10^5^	31.9% CFU	no	183−304 μm	wild-type like
***hmbAΔ veA***^***+***^	delayed compared to wild-type	estimated 0−10	estimated 30% germinating	yes	166−270 μm	conditional**
***hmbBΔ veA***^***+***^	delayed compared to wild-type	conditional* 4×10^4^ ±2.6×10^4^	0.04% CFU	yes	conditional* 200−225 μm	conditional*at the perimeter of the colonies
***hmbCΔ veA***^***+***^	wild-type	2×10^5^ ±7.3×10^4^	6.9% CFU	yes	195−354 μm	wild-type like

* only upon medium level of oxygen restriction

** wild-type like distribution pattern in sealed plates; exclusive central accumulation in non-sealed plates

The control *veA*^*+*^ strain formed primordia on day 5 (image A on Fig 1) and formed mature ascospores on day 10 of incubation (image B on Fig 1). The *veA1* control developed more rapidly than the *veA*^*+*^ control and μ-cleistothecia formation was observed on day 5 of incubation (image C on Fig 1) and mature free ascospores were present from day 6 of incubation (image D on Fig 1).

The time course of the sexual development of the *hmbCΔ veA*^*+*^ strain was not different from that of the *veA*^*+*^ control, however the primordium formation of the *hmbAΔ veA*^*+*^ and *hmbBΔ veA*^*+*^ strains was delayed by a day or two compared to that of the *veA*^*+*^ control (images E, K and L on Fig 1).

Development of the sexual structures in the *hmbAΔ veA1* and *hmbBΔ veA1* strains were found to occur earlier (1–2 days) than in their *veA*^*+*^ counterparts (images F and M on Fig 1), however the *veA1* background did not accelerate sexual development in the *hmbCΔ veA1* mutant. The primordium and μ-cleistothecia formation of *hmbCΔ veA1* were observed one day later compared to the *hmbCΔ veA*^*+*^ strain (image R on Fig 1).

The mature *hmbAΔ* cleistothecia, collected on days 11 and 12 of incubation, were practically empty in both the *veA*^*+*^ (images G and H on Fig 1) and *veA1* background (images I and J on Fig 1). These cleistothecia contained RGA material, nearly zero amounts of ascogenous hyphae and sporadically detected ascospores (magnified sections of images H and J on Fig 1). On day 12, the mature *veA*^*+*^ and *veA1 hmbBΔ* cleistothecia contained ascogenous hyphae, RGA material, a few asci in different maturation stages and a few matured, free ascospores (images N and O on Fig 1). Cleistothecia with somewhat improved quality and quantity of internal content could only be observed under highly specific conditions (detailed below). On day 12, the *veA*^*+*^ *hmbCΔ* cleistothecia still contained immature asci with transparent ascospores among the released, mature ascospores (image P on Fig 1). The *veA1 hmbCΔ* mutant frequently produced barren cleistothecia without any ascospores. However, when the *veA1 hmbCΔ* cleistothecia contained ascospores, these ascospores were always mature (image Q on Fig 1). RGA material was detected in both *veA*^*+*^ and *veA1 hmbCΔ* cleistothecia, but this was more pronounced in the *veA1* background.

**Fig 2:**
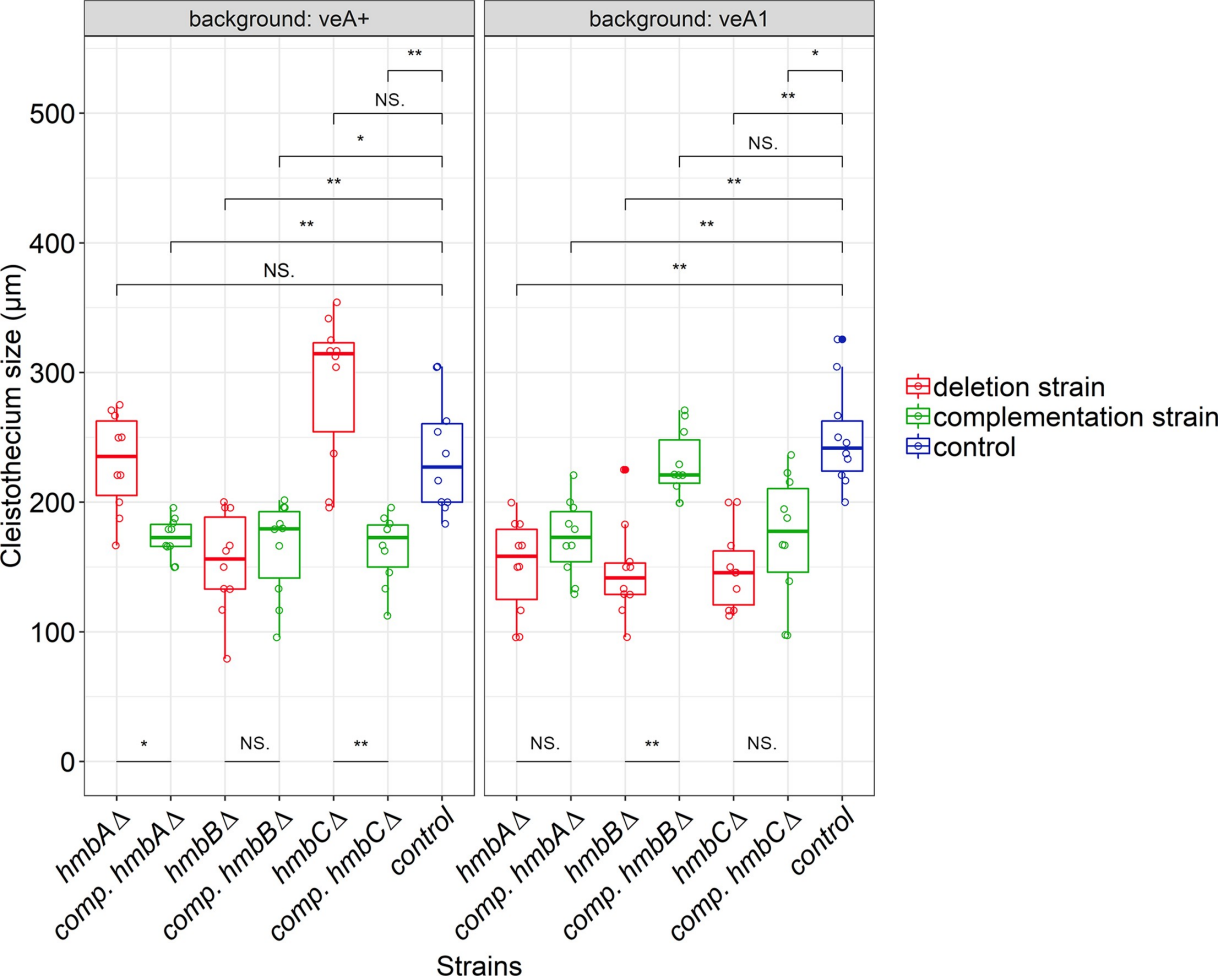
Size of cleistothecia in *hmbAΔ*, *hmbBΔ* and *hmbCΔ* mutants in *veA*^*+*^ and *veA1* genetic backgrounds. The boxplot shows the size of cleistothecia across the *hmbA*, *hmbB* and *hmbC* mutant and control strains in both the *veA*^*+*^ (left panel) and the *veA1* (right panel) genetic backgrounds. The strains are color-coded as follows: blue denotes control; red denotes deletion, green denotes complementation (comp.) strains originating from the corresponding deletion strains. Centre lines indicate the median of 10 independent cleistothecia measurements per strain. Mann-Whitney *U*-test was used to assess size differences between the mutant and control strains in the corresponding genetic background. */** indicates *p* < 0.01/0.001, NS indicates *p* = not significant. Cleistothecia sizes were estimated by measuring the diameter of cleistothecia with a ruler (for further details see S4 Fig). The strains used in the experiment are as follows: *veA*^*+*^ control (HZS.450), *veA1* control (HZS.145), *hmbAΔ veA*^*+*^ (HZS.521), *hmbAΔ veA1* (HZS.239), *hmbAΔ veA*^*+*^ with *hmbA* complementation (HZS.678), *hmbAΔ veA1* with *hmbA* complementation (HZS.621), *hmbBΔ veA*^*+*^ (HZS.495), *hmbBΔ veA1* (HZS.280), *hmbBΔ veA*^*+*^ with *hmbB* complementation (HZS.680), *hmbBΔ veA1* with *hmbB* complementation (HZS.677), *hmbCΔ veA*^*+*^ (HZS.531), *hmbCΔ veA1* (HZS.338), *hmbCΔ veA*^*+*^ with *hmbC* complementation (HZS.679), *hmbCΔ veA1* with *hmbC* complementation (HZS.676).

In the case of the colonies of *veA*^*+*^ and *veA1* controls, cleistothecia were accumulated in the middle of the colony and were equally distributed in the rest of the area regardless whether the plates were sealed or not (Fig 1). The size of the cleistothecia in *veA*^*+*^ and *veA1* controls was in the ranges of 183–304 μm and 216–304 μm, respectively (Fig 2, S4 Fig). The distribution pattern of the *hmbAΔ* cleistothecia was detected to depend on oxygen availability. Oxygen-deprivation (via sealing the plates) resulted in a wild type-like distribution pattern both in the *veA*^*+*^ and the *veA1 hmbAΔ* strains (Fig 1). However, when the plates were not sealed and thereby air exchange was not restricted, fruiting body formation was mainly exclusive to the central part of the colony in both the *veA*^*+*^ and the *veA1 hmbAΔ* strains (Fig 1). The size of cleistothecia in the *veA*^*+*^ and *veA1 hmbAΔ* strains was in the ranges of 166–270 μm and 95–200 μm, respectively (Fig 2, S4 Fig).

Selfing of the *hmbBΔ* strains (both *veA*^*+*^ and *veA1*) provided colonies with barren micro-sized cleistothecia and/or few small-sized cleistothecia (79–95 μm, Fig 2, S4 Fig) with a low ascospore content (~100 ascospores/cleistothecia) in nearly all cases. However, selfing plates with few normal-sized cleistothecia (up to 200–225 μm, Fig 2, S4 Fig) with an improved ascospore content were also detected sporadically. A systematic combination of environmental factors (light and oxygen availability) revealed that the *hmbBΔ* strain favors a medium-level of oxygen restriction. Neither the sealed, nor the non-sealed plates provided normal-sized cleistothecia. We found that the height of the medium (40–45 ml) in the Petri dish (9 cm diameter) combined with keeping the plates unsealed was a determinant factor of normal-sized cleistothecia. Based on this phenomenon, we propose that the *hmbBΔ* strain needs a certain level of oxygen for the optimal sexual development in both the *veA*^*+*^ and the *veA1* backgrounds. The distribution of sterile micro-sized cleistothecia resembled the wild type, however when the environmental parameters favored the production of normal-sized cleistothecia, they were always formed at the perimeter of the colonies (Fig 1).

The distribution and the size of the *veA*^*+*^ *hmbCΔ* cleistothecia were similar to that of the *veA*^*+*^ control (195–354 μm), whilst *veA1 hmbCΔ* produced equally-distributed, medium-sized (112–200 μm) cleistothecia in lower abundance compared to its *veA*^*+*^ counterpart (Fig 1, Fig 2 and S4 Fig). The *veA1 hmbCΔ* colonies tended to form conidia-depleted sectors, where the density of cleistothecia was significantly increased (shown in S5 Fig and indicated in Fig 1).

The size of cleistothecia was restored only in the *veA1 hmbB* complemented strain (Fig 2). None of the other reconstitutions provided any improvement (*veA*^*+*^ *hmbB*, *veA1 hmbA* and *veA1 hmbC*) or even decreased (*veA*^*+*^ *hmbA* and *veA*^*+*^ *hmbC*) the size of cleistothecia (Fig 2), most probably due to the *in trans* expression of the cognate genes and/or the usage of a constitutive promoter in the case of *hmbB* and *hmbC* reconstitution.

### Ascospore content of cleistothecia and viability of ascospores in *veA*^*+*^ background

**Fig 3:**
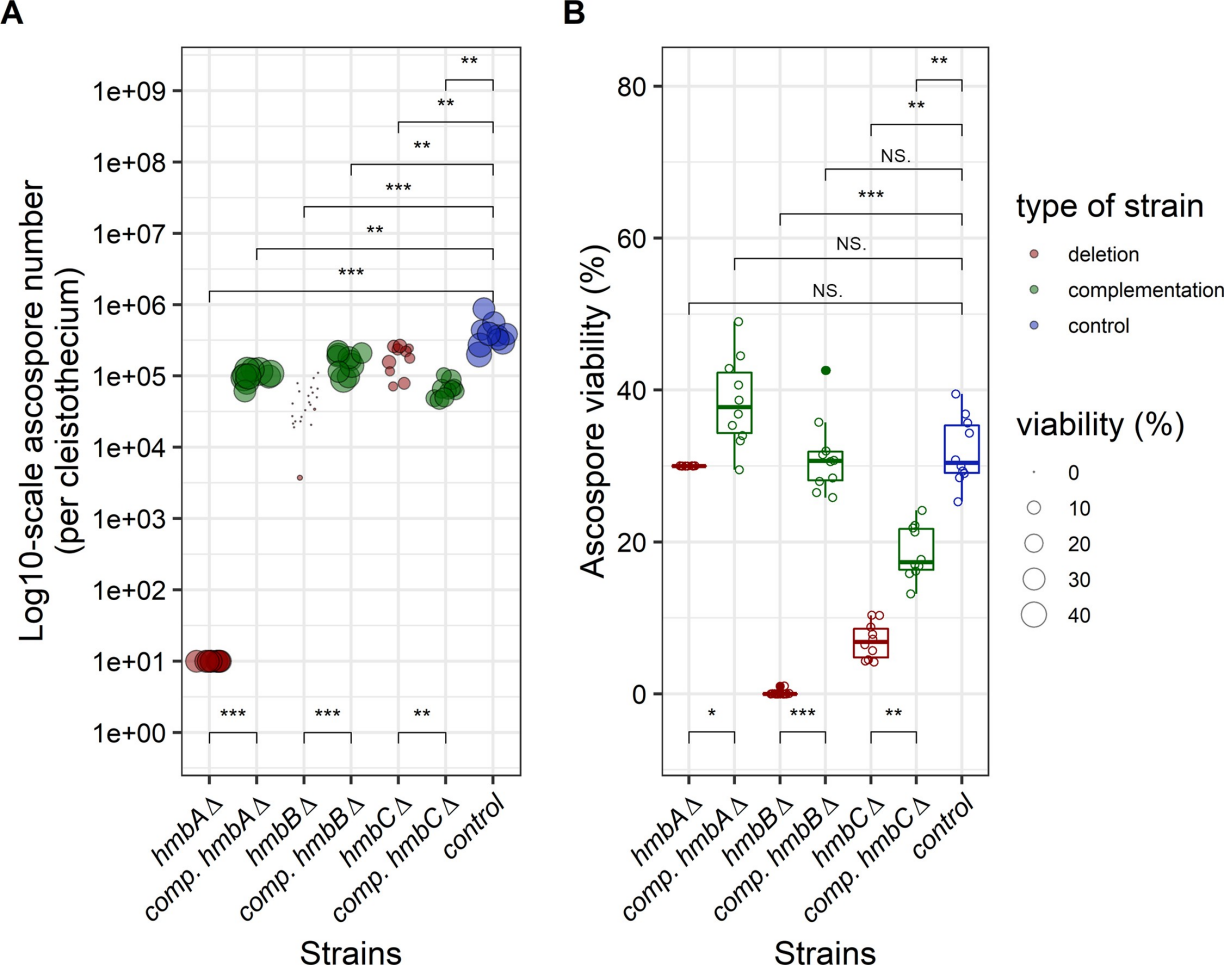
Production and viability of ascospores produced by *hmbAΔ*, *hmbBΔ* and *hmbCΔ* mutants in *veA*^*+*^ genetic background. Panel A: Graphical representation of the number of ascospores per cleistothecium. The figure shows the number of ascospores (on log_10_-scale) per cleistothecium across the *hmbA*, *hmbB* and *hmbC* mutant and control strains in a *veA*^+^ genetic background. The strains are color-coded as follows: blue denotes control; red denotes deletion, green denotes complementation (comp.) strains originating from the corresponding deletion strains. The bubbles mark the number of ascospores; the size of the bubbles is proportional to the viability of the corresponding ascospores. An estimated rate of germination was used as a proxy of viability in the case of *hmbAΔ* (explained in the main text). The number of ascospores were estimated based on 10 independent cleistothecia per strain. An estimated ascospore number was used in the case of *hmbAΔ* (explained in the main text). Mann-Whitney *U*-test was used to assess the differences between the numbers of ascospores of the mutant and the control strains. */**/*** indicates *p* < 0.01/0.001/0.0001. Panel B: Graphical representation of the viability of ascospores. The boxplot shows the viability of the ascospores across the *hmbA*, *hmbB* and *hmbC* mutant and control strains in a *veA*^+^ genetic background. Viability was calculated by counting the number of colony-forming ascospores on solid medium. An estimated rate of germination was used as a proxy of viability in the case of *hmbAΔ* (explained in the main text). The strains are color-coded as follows: blue denotes control; red denotes deletion, green denotes complementation (comp.) strains originating from the corresponding deletion strains. Centre lines indicate the median viability of ascospores collected from 10 independent cleistothecia per strain. Mann-Whitney *U*-test was used to assess size differences between the mutant strains and the control in the corresponding genetic background. */**/*** indicates *p* < 0.01/0.001/0.0001, ns indicates *p* = not significant. The strains used in the experiment are as follows: *veA*^*+*^ control (HZS.450), *hmbAΔ veA*^*+*^ (HZS.521), *hmbAΔ veA*^*+*^ with *hmbA* complementation (HZS.678), *hmbBΔ veA*^*+*^ (HZS.495), *hmbBΔ veA*^*+*^ with *hmbB* complementation (HZS.680), *hmbCΔ veA*^*+*^ (HZS.531), *hmbCΔ veA*^*+*^ with *hmbC* complementation (HZS.679).

The *veA*^*+*^ control contained 10^5^–10^6^ ascospores per cleistothecium, and 32% of them were able to form colonies (Fig 3, S1 Data). We estimated the number of ascospores in the *hmbAΔ veA*^*+*^ cleistothecia (≤10 ascospores/cleistothecium) by counting the visible ascospores in crushed cleistothecia samples (Fig 3). The frequency of viable *hmbAΔ veA*^*+*^ ascospores could not be calculated based on counting the colony forming units (CFU), because the extremely low (≤10) number of CFUs might also originate from mycelium-, conidiospore- or Hülle cell contamination of the cleaned cleistothecium samples. We therefore decided to monitor the germination rate of the *hmbAΔ veA*^*+*^ ascospores using a microscope that does not necessarily reflect colony forming ability (viability). We collected and pooled 25 *hmbAΔ veA*^*+*^ cleistothecia, and the total content was incubated in liquid MM on the surface of cover slips. Remarkably, we found that at least 30% of these ascospores were viable (Fig 3), although we have no proof that the germinating ascospores can also form colonies. Complementation of the *hmbAΔ* deletion phenotype (≤10 ascospores per cleistothecium) by *in trans* expression of *hmbA* under the control of its native promoter resulted in ~10^5^ ascospores per cleistothecium (with 38% viability rate), which was significantly lower than that of the wild-type control, however, it approached the productivity level of the control.

The ascospore content of *hmbBΔ veA*^*+*^ cleistothecia increased with the age of the colony (10^3^ ascospores per cleistothecium at week 2, 10^4^ ascospores per cleistothecium at week 4 and 10^5^ ascospores per cleistothecium at week 6). However, the viability of these ascospores decreased with aging (1% at week 2, 0.06% at week 4 and 0% at week 6). Complementation of the *hmbBΔ* deletion phenotype (~4×10^4^ ascospores per cleistothecium) by *in trans* expression of *hmbB* under the control of the constitutive *gpdA* promoter resulted in an ascospore productivity approaching that of the wild type (~1.6×10^5^ ascospores per cleistothecium) with a viability rate of 31% (Fig 3). Even though ascospore productivity was significantly lower than that of the wild-type control, the difference was marginal. Ascospore productivity of the *hmbCΔ veA*^*+*^ strain was also similar to that of the wild type (~2×10^5^ ascospores/cleistothecium); however, only 7% of them was found to be viable (Fig 3). Complementation of the *hmbCΔ* deletion phenotype (~2×10^5^ ascospores per cleistothecium) by *in trans* expression of *hmbC* under the control of the constitutive *gpdA* promoter resulted in significantly decreased ascospore productivity (~6.3×10^4^ ascospores/cleistothecium) compared to that of the deleted strain, however the viability of the ascospores increased more than two-fold (18.6%) compared to that of the deletion strain (Fig 3).

### Mating-type MAT1-1 and MAT1-2 coding genes are extremely down-regualted in the *veA*^*+*^ *hmbAΔ*, *hmbBΔ and hmbCΔ* mutants

**Fig 4:**
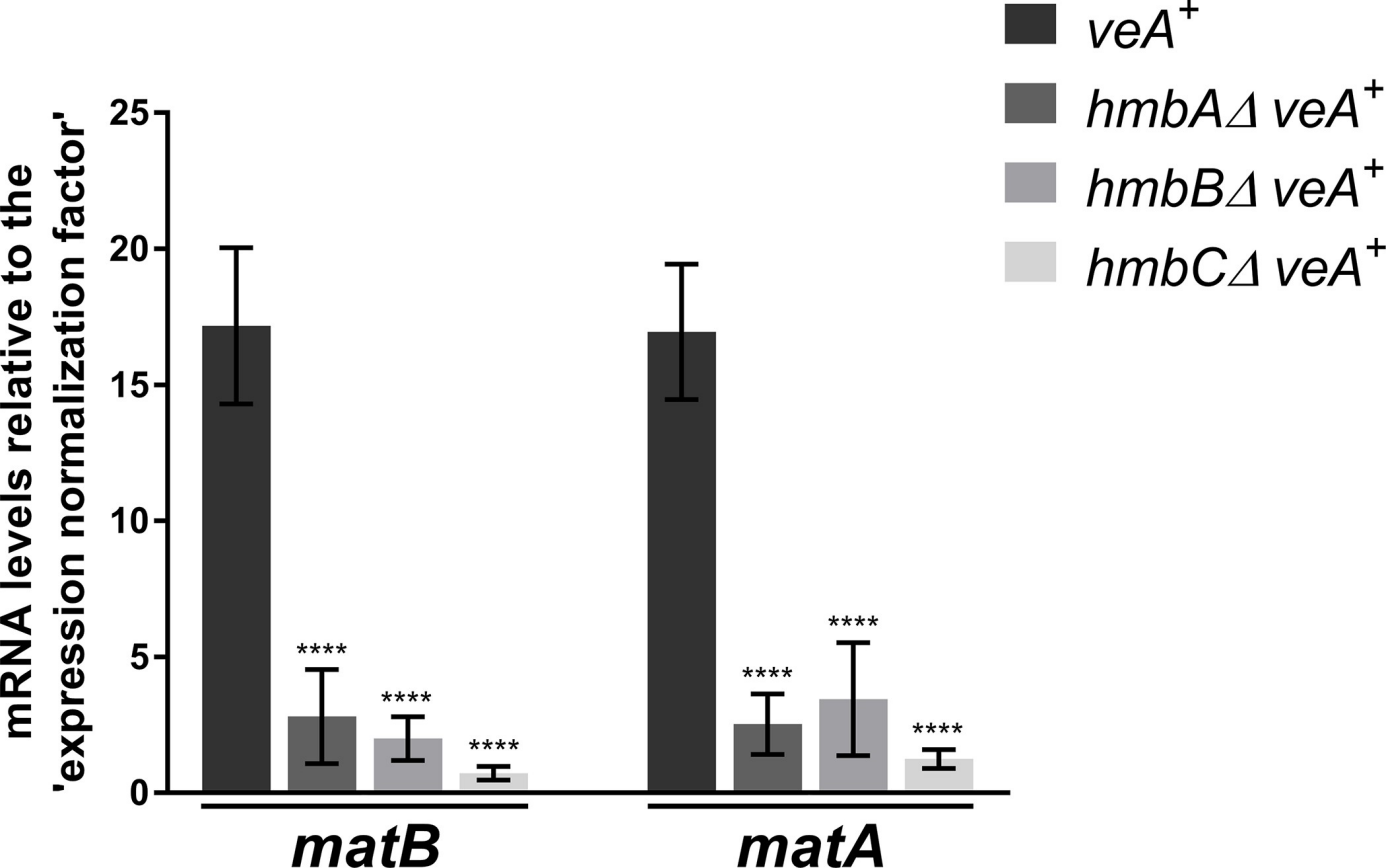
mRNA levels measured by qRT-PCR for MAT1-1 coding *matB* and MAT1-2 coding *matA* genes in *veA*^*+*^ control and *veA*^*+*^ *hmbAΔ*, *hmbBΔ* and *hmbCΔ* strains 96 h after the induction of sexual development. Results, obtained by calculations according to the standard curve method [47], were normalized to an ‘expression normalization factor’ calculated from two selected reference genes (*tubC* and *gpdA*) (detailed in S1 Materials and methods). Standard deviations of three technical replicates of three biological samples are shown. The stars above the columns indicate the significance of the differences compared to the *veA*^*+*^ control. Significant differences between the mutants and the control were determined by using a two-way ANOVA test. **** indicates *p* < 0.0001. The following strains were used in the experiment: *veA*^*+*^ (HZS.450), *hmbAΔ veA*^*+*^ (HZS.521), *hmbBΔ veA*^*+*^ (HZS.495) and *hmbCΔ veA*^*+*^ (HZS.531). The cultivation settings were as follows: approximately 10^6^ conidiospores per strain were inoculated into liquid MM, and were grown for 24 hours at 37°C with 180 rpm shaking. Then the vegetatively grown mycelia were transferred onto solid MM covered with cellophane, sealed carefully with scotch tape and incubated for 96 hours at 37°C in complete darkness. After the incubation period, total RNA was extracted and processed.

The expression of the mating-type MAT1-1 and MAT1-2 coding *matB* and *matA* genes starts at the late stage of sexual development (four days after the induction of sexual development), when Hülle cells and immature cleistothecia are already formed [35]. We determined the mRNA levels of *matB* and *matA* on day 4 of sexual development (96 h after the induction of sexual development) and found that both mating-type genes were extremely down-regulated in all of the deletion mutants (showing a 4.9–23.6-fold negative change) (Fig 4). As discussed in details below, this extremely high degree of down-regulation might indicate the aberrant ascospore development in cleistothecia observed in the mutants.

## Discussion

According to the results presented above, HmbA, HmbB and HmbC architectural chromatin components of *A*. *nidulans* are required for normal sexual development, especially for the formation and viability of ascospores and the spatial distribution of cleistothecia (Table 2, S1 Table). By studying *hmbA*, *hmbB* and *hmbC* deletions in both *veA*^*+*^ and *veA1* background, we have investigated the possible functional interaction of these HMGB proteins with VeA, the master regulator of sexual development (Table 2, S1 Table).

**Table 2:** Summary of detected functions of HmbA, HmbB and HmbC.

role	HmbA	HmbB	HmbC
**direct and/or indirect role in positive regulation of MAT genes**	yes	yes	yes
**functional interaction with VeA**	not detected	not detected	yes
**involved in sensing of and responding to the environmental oxygen level**	yes	yes	no
**required for normal intracolonial distribution of cleistothecia**	yes, conditionally (oxygen-deprivation)	yes, conditionally (medium-level of oxygen-deprivation)	no
**required for normal time course of sexual development**	yes	yes	yes, VeA-dependently
**required for normal abundance of cleistothecia**	yes	yes	yes, VeA-dependently, along with sectoring
**role in ascospore productivity**	pivotal	important	pivotal, VeA-dependently
**role in ascospore viability**	not confirmed*	pivotal	important

* 30% of ascospores are able to germinate, however their colony forming ability was not confirmed

The *hmbAΔ*, *hmbBΔ* and *hmbCΔ* strains were found to show defect in ascospore production and viability to various extent (Table 2). The *hmbA* deletion resulted in nearly sterile cleistothecia with less than 10 ascospores inside, which were remarkably able to germinate with an estimated 30% rate, however the colony forming ability of these germinating ascospores was not confirmed. The deletion of *hmbB* and *hmbC* resulted in a decreased ascospore productivity, accompanied by a severe (in *hmbBΔ*) and a less-severe (in *hmbCΔ*) decrease of ascospore viability. RGA material was detected in all three deleted mutants; amongst them the *hmbCΔ* strain accumulated RGA material to a greater extent, which was more pronounced in the *veA1* background. The *veA*^*+*^ *hmbCΔ* cleistothecia invariably contained asci and ascospores, however, these asci were frequently immature. Remarkably, the *veA1 hmbCΔ* cleistothecia were either barren (devoid of ascospores) or fertile (containing ascospores). The fertile *veA1 hmbCΔ* cleistothecia mostly contained mature, free ascospores and an increased amount of RGA material. Furthermore, the abundance of the *hmbCΔ* cleistothecia approached that of the wild type in the *veA*^*+*^ background, whereas it was reduced in the *veA1* background. Both *hmbA* and *hmbB* deletions caused a delay in the time course of sexual development, independently of VeA, while *hmbC* deletion had no effect on sexual development in the *veA*^*+*^, but caused a pronounced delay in the *veA1* background. Considering all the above described *hmbCΔ* phenotypes, we propose that HmbC functionally interacts with VeA (Table 2, S1 Table).

HmbA and HmbB might be involved in sensing of and responding to the changes of environmental oxygen levels. The wild type-like distribution of the cleistothecia was found to depend on the strict deprivation of oxygen in the *hmbAΔ* mutants, while normal-sized cleistothecia formation in the *hmbBΔ* strain required a medium level of oxygen deprivation (Table 2, S1 Table).

Mating-type protein functions in heterothallic fungi are essential for pheromone-signalling and recognition of the mating partner [48, 49]. However, mating-type proteins of the homothallic *A*. *nidulans* (MAT1-1 encoded by *matB* and MAT1-2 encoded by *matA*) are essential for the fertility of the formed cleistothecia by contributing to ascospore production [35]. Deletion of *matA* or *matB* does not affect Hülle cell formation and cleistothecium production, however the cleistothecia are devoid of ascospores and contain only granular amorphous materials [35]. The MAT gene deletion phenotype is somewhat similar to the ascospore production- and viability-related phenotypes of the *hmbA*, *hmbB* and *hmbC* deleted strains. As expected, HmbA, HmbB and HmbC were found to be critical for the normal expression of the MAT genes on day 4 after the initiation of sexual development (Fig 4, Table 2). We excluded the possibility that all three HMGB linker proteins are required for the direct activation of MAT gene expression. Instead, we propose an alternative explanation for the observed changes: HmbA, HmbB and HmbC might operate on upstream MAT gene regulator(s) that is (are) most probably involved in the sensing of environmental and/or intracellular factors and/or the transduction of related signals that affect the activation of the MAT genes. Future research should elucidate the potential role of HMGB proteins in the upstream regulation of MAT genes. HmbA, HmbB and HmbC orthologue proteins of the heterothallic fungus *P*. *anserina* (PaHMG6, mtHMG1 and PaHMG4, respectively) do not contribute equally to the regulation of mating-type transcription factors (coded by *fmr1* and *fpr1*, and being essential for the regulation of mating-type specific genes and the recognition of the mating partner at the pheromone-signalling stage) [48]. Only the HmbA orthologue protein, PaHMG6 regulates positively *fmr1* in a mat^−^strain (in a mat^+^ strain the regulation bypasses *fpr1*), while the MAT genes are negatively regulated by the HmbB orthologue protein mtHMG1 and unaffected by the HmbC orthologue protein PaHMG4 [33] (S1 Table).

Although all three HMGB proteins play a role in the expression of the mating-type genes, neither the *hmbB*, nor the *hmbC* deletion phenocopied the *matBΔ* or *matAΔ* phenotypes completely. The deletion phenotypes of *hmbBΔ* (both *veA*^*+*^ and *veA1*) and *veA*^*+*^ *hmbCΔ* resembled to that of the *matBΔ* or *matAΔ* regarding RGA material accumulation inside the cleistothecia, however, ascospores were produced, although with deficient viability. Only the *hmbAΔ* (in both *veA*^*+*^ and *veA1*) phenotype shared all the characteristics of the *matBΔ* and *matAΔ* phenotypes. The MAT gene deletion phenotype was frequently, but not always observed in the *veA1 hmbCΔ* strain, which indicates that HmbC functionally interacts with VeA. On the other hand, the random occurrence of barren cleistothecia amongst fertile cleistothecia might reflect a sporadic compensation for the loss of *hmbC* in the *veA1* background. We cannot exclude the possibility that HmbA, HmbB and HmbC directly influence the gene expression of MAT-regulated genes, and that they can provide the functional backups for each other’s loss. Such a compensatory effect had already been reported in the case of other types of linker proteins. Mice contain eight subtypes of the linker H1 histone that are differentially expressed during development [50–52]. These H1 histone variants can compensate for each other’s loss in homozygous knockout mouse models (e.g. H1c, H1d and H1e variants can compensate for the homozygous deletion of H1(0)) [53].

Considering all of the phenotypes of the *hmbAΔ*, *hmbBΔ* and *hmbCΔ* strains, it is reasonable to suppose that besides the pronounced down-regulation of MAT genes, a qualitatively different perturbation in the expression of other genes might further contribute to the *hmbAΔ-*, *hmbBΔ-* and *hmbCΔ-*specific phenotypes, however, investigation of these changes are beyond the scope of this work.

Based on the orthologous relation of HmbA, HmbB and HmbC proteins with PaHMG6, mtHMG1 and PaHMG4 proteins of *P*. *anserina*, respectively, we compared the roles of the orthologous proteins to assess functional relations (S1 Table). The orthologue of HmbA in *P*. *anserina*, PaHMG6, is required for achieving a normal-sized colony [33]. This function is qualitatively similar to that seen in the case of HmbA of *A*. *nidulans* (S3 Fig). In a homozygous cross, the *Pahmg6Δ* mutant produced fruiting bodies with 50 times less abundance, with smaller body and larger neck compared to the wild type, and began to eject ascospores several days later in comparison to the wild type. Although both *A*. *nidulans hmbAΔ* and *P*. *anserina Pahmg6Δ* mutants showed a delay in the time course of sexual development in homozygotic crosses, an analogy between their functions in the aspect of sexual competency cannot be concluded.

The absence of the orthologue of HmbB in *P*. *anserina AS1*^*+*^ strain (*mthmg1Δ*, *AS1*^*+*^) does not result in the loss of the ascospores’ ability for germination (ascospores germinate slowly with a spindly phenotype [12]), thus we cannot establish an analogy between the functions of mtHMG1 and HmbB. Although the dual localization of the mtHMG1 protein of *P*. *anserina* has not been studied yet, we previously revealed a dual localization of HmbB, and found that the orthologous HmbB and mtHMG1 share a third HMG-box domain, called Shadow-HMG-box, which is characteristic to the HmbB orthologues across Pezizomycotina [11]. The structural similarity between HmbB and mtHMG1, as well as the fact that both proteins modulate the expression of nuclear genes [11, 33] makes it reasonable to suppose that mtHMG1 fulfils nuclear-localization-linked functions as we suggested previously for HmbB [11].

The HmbC counterpart in *P*. *anserina*, PaHMG4, was found to be required for the normal distribution of the fruiting bodies [33]. The PaHMG4 deletion mutant produced five times more spermatia (with wild type-like viability), whereas the deletion had no effect on female fertility [33]. Thus, PaHMG4 function differs from that of HmbC, thereby the two proteins are functionally diverged.

Some of the physiological functions of HMGB proteins we revealed are specific for *A*. *nidulans* compared to yeast and *P*. *anserina* (summarized in S1 Table). This includes that HmbA and HmbB play a role in sensing of and/or response to environmental signals. By revealing the functional connections of HmbA and HmbB with signal transduction pathways, one would gain a deeper understanding of the regulatory machinery that governs physiological responses to environmental changes. On the other hand, we found that HmbC functionally interacts with VeA, a key regulator of the coordination of asexual and sexual development, as well as of secondary metabolism. By revealing the functional interactions of HmbC, one would gain a deeper insight into the regulation of these biological processes. Finally, HmbA, HmbB and HmbC are equally important in the positive regulation of mating-type genes, and thereby have a great impact on ascospore production in *A*. *nidulans*. The knowledge on the regulation of fungal mating-type genes is scarce, thereby clarifying, whether these HMGB proteins influence *matA*/*matB* expression directly or indirectly (e.g. via the modulation of upstream regulatory factors) would be of great interest. Additionally, future works should elucidate the gene-expression modulatory role of the HMGB proteins on a genome-scale that might lead to a more detailed characterization of the physiological roles of HmbA, HmbB and HmbC.

## Supporting information

S1 Table Physiological functions of yeast architectural HMGB proteins and their orthologue counterparts from *P. anserina* and *A. nidulans*. (PDF)

S2 Table *A. nidulans* strains used in this work. (PDF)

S3 Table Used primers. (PDF)

S1 Materials and methods 1. Construction of reconstitution vectors. 2. RT-qPCR. (PDF)

S1 Data Ascospore number and viability; Standard curves, result of geNORM analysis, Gene expression data set. (XLSX)

S1 Fig Verification of single copy integration events in *hmbA* deletion transformants by Southern analysis. Panel A. Schematic representation of the genomic region of *hmbA*^*+*^. The red and blue segments represent the sequence regions used to target the genomic regions by homologous recombination (HR1: homologous recombination sequence upstream to the deletion target, HR2: homologous recombination sequence downstream to the deletion target). The total DNAs of the *hmbA*^*+*^ control strain and putative deleted transformants were digested with XbaI restriction endonuclease. Zig-zag arrows show the positions of the XbaI cleavage sites. The Southern blot of XbaI digested total DNAs was probed with a digoxigenine labelled PCR product, as indicated in the scheme ("Probe"). Green and yellow boxes indicate the targeted *hmbA* gene and the *riboB*^*+*^ selection marker gene used for the gene-substitution, respectively. Arrows show the size of the hybridizing DNA fragments obtained by XbaI digestion.
Panel B. Schematic representation of the substitution cassette constructed by the Double-Joint PCR method [40] (carrying the *riboB*^*+*^ selection marker gene) at the bottom of the panel and the arrangement of the targeted genomic region after the gene substitution event (by double cross overs between HR1 and HR2 regions) at the top of the panel. Dashed lines indicate homologous recombination events. Zig-zag arrows show the positions of the XbaI cleavage sites in the gene-substituted genomic region.
Panel C. Image of the Southern hybridisation filter showing the *hmbA*^*+*^ signal on the left and the *hmbAΔ* signal on the right. The *hmbA*^*+*^ strain is the recipient parent HZS.120 and the presented deletion mutant is the HZS.205.
(PDF)

S2 Fig Verification of single copy integration events in *hmbC* deletion transformants by Southern analysis. Panel A. Schematic representation of the substitution cassette constructed by the Double-Joint PCR method [40] (carrying the *pabaA*^*+*^ selection marker gene) at the bottom of the panel and the arrangement of the targeted genomic region after the gene substitution event (by double cross overs between HR1 and HR2 regions) at the top of the panel. The red and blue segments represent the sequence regions used to target the genomic regions by homologous recombination (HR1: homologous recombination sequence upstream to the deletion target, HR2: homologous recombination sequence downstream to the deletion target). Yellow box mark the *pabaA*^*+*^ selection marker gene. The total DNAs of the *hmbC*^*+*^ control strain and putative deleted transformants were digested with EcoRV restriction endonuclease. Zig-zag arrows show the positions of the EcoRV cleavage sites in the gene-substituted genomic region. The Southern blot of EcoRV digested total DNAs was probed with a digoxigenine labelled PCR product, as indicated in the scheme ("Probe"). Dashed lines indicate homologous recombination events.
Panel B. Schematic representation of the genomic region of *hmbC*^*+*^. Green box indicate the targeted *hmbC* gene. Zig-zag arrows show the positions of the EcoRV cleavage sites. Arrows show the size of the hybridizing DNA fragments obtained by EcoRV digestion.
Panel C. Image of the Southern hybridisation filter showing the *hmbC*^*+*^ signal on the left and the *hmbCΔ* signal on the right. The *hmbC*^*+*^ strain is the recipient parent HZS.120 and the presented deletion mutant is the HZS.338.
(PDF)

S3 Fig Growth ability of *veA*^+^ and *veA1* controls, *hmbAΔ*, *hmbBΔ* and *hmbCΔ* strains and their cognate complementation (reconstitution) strains in both *veA*^+^ and *veA1* background. The strains were incubated on CM for 2 days at 37°C. Strains used: *veA*^*+*^ control (HZS.450), *veA1* control (HZS.145), *hmbAΔ veA*^*+*^ (HZS.521), *hmbAΔ veA1* (HZS.239), *hmbBΔ veA*^*+*^ (HZS.495), *hmbBΔ veA1* (HZS.280), *hmbCΔ veA*^*+*^ (HZS.531), *hmbCΔ veA1* (HZS.338), *hmbAΔ veA*^*+*^ *hmbA* reconstituted (HZS.678), *hmbAΔ veA1 hmbA* reconstituted (HZS.621), *hmbBΔ veA*^*+*^ *hmbB* reconstituted (HZS.680), *hmbBΔ veA1 hmbB* reconstituted (HZS.677), *hmbCΔ veA*^*+*^ *hmbC* reconstituted (HZS.679), *hmbCΔ veA1 hmbC* reconstituted (HZS.676). The complete genotypes are listed in S2 Table.
(PDF)

S4 Fig Comparison of cleistothecia-sizes produced by *veA*^+^ and *veA1* controls, *hmbAΔ*, *hmbBΔ* and *hmbCΔ* strains and their cognate complementation (reconstituted) strains in both *veA*^+^ and *veA1* background. Cleistothecia were collected from colonies, and subsequently purified by rolling them on a sterile agar plate and documented by a camera in the presence of a ruler. Scale bar on the figure shows 1000 μm. Strains used: *veA*^*+*^ control (HZS.450), *veA1* control (HZS.145), *hmbAΔ veA*^*+*^ (HZS.521), *hmbAΔ veA1* (HZS.239), *hmbBΔ veA*^*+*^ (HZS.495), *hmbBΔ veA1* (HZS.280), *hmbCΔ veA*^*+*^ (HZS.531), *hmbCΔ veA1* (HZS.338), *hmbAΔ veA*^*+*^ *hmbA* reconstituted (HZS.678), *hmbAΔ veA1 hmbA* reconstituted (HZS.621), *hmbBΔ veA*^*+*^ *hmbB* reconstituted (HZS.680), *hmbBΔ veA1 hmbB* reconstituted (HZS.677), *hmbCΔ veA*^*+*^ *hmbC* reconstituted (HZS.679), *hmbCΔ veA1 hmbC* reconstituted (HZS.676). The complete genotypes are listed in S2 Table.
(PDF)

S5 Fig Sectoring of *veA1 hmbCΔ* colonies. Strain HZS.338 was incubated on CM for 4 days at 37°C. Sectoring areas are magnified.
(PDF)
