# Supplementary material for: HMGB proteins are required for sexual development in Aspergillus nidulans
Source: PLoS One. 2019 Apr 25;14(4):e0216094. doi: 10.1371/journal.pone.0216094 (PMC6483251; doi:10.1371/journal.pone.0216094)
Supplement: S1 Table — (PDF) [file pone.0216094.s001.pdf]

S1 Table. Physiological functions of yeast architectural HMGB proteins and their orthologue counterparts from *P. anserina* and *A. nidulans*.

**role of HMGB proteins**	***S. cerevisiae* (Sc)**	***P. anserina* (Pa)**	***A. nidulans* (An)**
**Nhp6A/Bp (Sc) and its orthologue PaHMG6 (Pa) and HmbA (An)**			
role in normal growth at high temperature	yes [1]	N.S.	N.S.
role in normal distribution of cytoskeletal elements	yes [1]	N.S.	N.S.
role in surviving under N-starvation	yes [1]	N.S.	N.S.
role in sexual reproduction	no [1]	yes [2]	yes (this work)
required for positive regulation of MAT genes	N.S.	yes, regulates *fmr1* in a mat⁻ strain [2]	yes (this work)
required for normal-sized colony	N.A.	yes [2]	yes (this work)
required for fruiting body formation	N.A.	no [2]	no (this work)
role in normal time course of sexual development	no [1]	yes [2]	yes (this work)
required for normal abundance of fruiting bodies	N.A.	yes [2]	yes (this work)
required for normal distribution of fruiting bodies	N.A.	no [2]	yes under oxygenated conditions (this work)
required for normal-sized fruiting bodies	N.A.	yes [2]	yes (this work)
required for ascospore formation	no [1]	N.S.	yes (this work)
role in sensing and/or response to environmental signals	N.S.	N.S.	yes (this work)
**Hmo1p (Sc) and its orthologue PaHMG4 (Pa) and HmbC (An)**			
required for normal-sized colony	yes [3]	no [2]	no (this work)
required for fruiting body formation	no [3]	no [2]	no (this work)
required for ascospore formation	no [3]	no [2]	yes (this work)
role in normal time course of sexual development	no [3]	no [2]	yes VeA dependently (this work)
required for distribution of fruiting bodies	N.A.	yes [2]	no (this work)
role in ascospore viability (colony forming ability)	no [3]	no [2]	yes (this work)

required for positive regulation of MAT genes	N.S.	no [2]	yes (this work)
functionally interacts with VeA	N.A.	N.A.	yes (this work)
**Hmo2p (Sc) and its orthologue KEF1 (Pa).** Orthologue in *A. nidulans* was not found.			
required for double strand break repair	yes [4]	N.S.	N.A.
repression of hyphal/cell anastomoses	N.S.	yes [2]	N.A.
**Abf2p (Sc) and its orthologues, mtHMG1 (Pa) and HmbB (An)**			
required for maintenance of mitochondrial genome	yes (on fermentable C-source) [5]	yes [6]	yes [7]
role in recombination	yes [8]	yes (partial suppression of premature death in an AS1-4 context by an extra copy of *mthmg1*) [6]	N.S.
role in ascospore viability (colony forming ability)	no [5]	N.S.	yes [7]
role in germination of ascospores	no [5]	yes slow germination with a spindly phenotype in *mthmg1Δ* in an $AS1^+$ backgorund [6]	yes ([7] and this work)
role in viability of vegetative spores	N.A.	N.A.	yes [7]
required for fruiting body formation	N.A.	yes contributes to perithecium formation and hymenium development [2]	no ([7] and this work)
required for ascospore productions	no [5]	N.S.	yes ([7] and this work)
required for normal distribution of fruiting bodies	N.A.	no [2]	yes (this work)
role in sensing and/or response to environmental signals	N.S.	N.S.	yes (this work)
required for positive regulation of MAT genes	N.S.	no [2]	yes (this work)
required for negative regulation of MAT genes	N.S.	yes [2]	no (this work)
role in life span	no	yes, life span is reduced in *mthmg1Δ* in an $AS1^+$ backgorund [6]	no [7]
required for sterigmatocystin production	N.A.	N.A.	yes [7]
required for maintenance of redox homeostasis	N.S.	N.S.	yes [9]
mitochondial and nuclear co-localization	no	N.S.	yes [7]
required for the expression of	no	N.S.	yes ([7,9] and

functionally diverse nuclear genes			this work)

N.A.: not applicable
N.S.: not studied
colored cells: mark those functions that are particular for *A. nidulans* HMGB proteins. Blue color marks functions identified in this work, while green color marks earlier reported functions.

References:

1. Costigan C, Kolodrubetz D, Snyder M (1994) NHP6A and NHP6B, which encode HMG1-like proteins, are candidates for downstream components of the yeast SLT2 mitogen-activated protein kinase pathway. Mol Cell Biol 14: 2391-2403.
2. Ait Benkhali J, Coppin E, Brun S, Peraza-Reyes L, Martin T, et al. (2013) A network of HMG-box transcription factors regulates sexual cycle in the fungus *Podospora anserina*. PLoS Genet 9: e1003642.
3. Lu J, Kobayashi R, Brill SJ (1996) Characterization of a high mobility group 1/2 homolog in yeast. J Biol Chem 271: 33678-33685.
4. Morrison AJ, Highland J, Krogan NJ, Arbel-Eden A, Greenblatt JF, et al. (2004) INO80 and gamma-H2AX interaction links ATP-dependent chromatin remodeling to DNA damage repair. Cell 119: 767-775.
5. Diffley JF, Stillman B (1991) A close relative of the nuclear, chromosomal high-mobility group protein HMG1 in yeast mitochondria. Proc Natl Acad Sci U S A 88: 7864-7868.
6. Dequard-Chablat M, Allandt C (2002) Two copies of mthmg1, encoding a novel mitochondrial HMG-like protein, delay accumulation of mitochondrial DNA deletions in *Podospora anserina*. Eukaryot Cell 1: 503-513.
7. Karacsony Z, Gacser A, Vagvolgyi C, Scazzocchio C, Hamari Z (2014) A dually located multi-HMG-box protein of *Aspergillus nidulans* has a crucial role in conidial and ascospore germination. Mol Microbiol 94: 383-402.
8. MacAlpine DM, Perlman PS, Butow RA (1998) The high mobility group protein Abf2p influences the level of yeast mitochondrial DNA recombination intermediates in vivo. Proc Natl Acad Sci U S A 95: 6739-6743.
9. Karacsony Z, Gacser A, Vagvolgyi C, Hamari Z (2015) Further characterization of the role of the mitochondrial high-mobility group box protein in the intracellular redox environment of *Aspergillus nidulans*. Microbiology 161: 1897-1908.
