# Supplementary material for: HMGB proteins are required for sexual development in Aspergillus nidulans
Source: PLoS One. 2019 Apr 25;14(4):e0216094. doi: 10.1371/journal.pone.0216094 (PMC6483251; doi:10.1371/journal.pone.0216094)
Supplement: S2 Table — (PDF) [file pone.0216094.s002.pdf]

S2 Table. *A. nidulans* strains used in this work.

Strain	Genotype	Purpose	Reference
		veA1 strains	
CS2902	*pyrG89 riboB2pyroA4 biA1*	parental strain in genetic crosses	[1]
HZS.98	*pantoB100 pabaA1 veA1*	parental strain in genetic crosses	this work
HZS.119	*anA1 riboB2 yA2 veA1*	parental strain in genetic crosses	this work
HZS.120	*riboB2 pabaA1 veA1*	recipient strain in transformation experiment	[2]
HZS.145	*veA1*	studies of sexual structure, RT-qPCR	this work
HZS.205	*hmbAΔ::riboB*$^+$ *riboB2 pabaA1 veA1*	parental strain in genetic crosses	this work
HZS.212	*hmbBΔ::riboB*$^+$ *riboB2 pabaA1 veA1*	parental strain in genetic crosses	[3]
HZS.227	*riboB2 pantoB100 yA2 veA1*	parental strain in genetic crosses	this work
HZS.239	*hmbAΔ::riboB*$^+$ *riboB2 veA1*	studies of sexual structure, RT-qPCR	this work (obtained by cross of HZS.205 with CS2902)
HZS.280	*hmbBΔ::riboB*$^+$, *yA2, veA1*	studies of sexual structure	this work (obtained by cross of HZS.212 with HZS.119)
HZS.314	*riboB2 pantoB100 pabaA1 biA1 veA1*	recipient strain in transformation experiment	this work
HZS.318	*hmbBΔ::riboB*$^+$ *riboB2 pabaA1 pantoB100 veA1*	recipient strain in transformation experiment	this work (obtained by cross of HZS.212 with HZS.227)
HZS.320	*hmbAΔ::riboB*$^+$ *riboB2 pabaA1 pantoB100 veA1*	recipient strain in transformation experiment	this work (obtained by cross of HZS.205 with HZS.227)
HZS.324	*pantoB100 riboB2 biA1 veA1*	parental strain in genetic crosses	this work
HZS.334	*hmbAΔ::riboB*$^+$ *hmbBΔ::riboB*$^+$ *riboB2 pabaA1 veA1*	parental strain in genetic crosses	this work
HZS.338	*hmbCΔ::pabaA*$^+$ *pabaA1 riboB2 pantoB100 biA1 veA1*	parental strain in genetic crosses, recipient strain in transformation experiment	this work
HZS.621	*hmbAΔ::riboB*$^+$ *riboB2 pantoB100 pabaA1 veA1* and 1 copy integration of pAN-HZS-9 containing *hmbA*$^+$	reconstituted *hmbAΔ* strain for studies of sexual structure	this work (obtained by transformation of pAN-HZS-9 into recipient strain HZS.320)
HZS.676	*hmbCΔ::pabaA*$^+$ *pabaA1 riboB2 pantoB100 biA1 veA1* and 1 copy integration of pAN-HZS-10 containing *hmbC*$^+$	reconstituted *hmbCΔ* strain for studies of sexual structure	this work (obtained by transformation of pAN-HZS-10 into recipient strain HZS.338)
HZS.677	*hmbBΔ::riboB*$^+$ *riboB2 pabaA1 pantoB100 veA1* and 1 copy integration of pAN-HZS-11 containing *hmbB*$^+$	reconstituted *hmbBΔ* strain for studies of sexual structure	this work (obtained by transformation of pAN-HZS-11 into recipient strain HZS.318)
		veA$^+$ strains	
HZS.450	*riboB2*	parental strain in genetic crosses, studies of sexual structure, RT-	this work

		qPCR	
HZS.451	*pabaA1 anA1 yA2*	parental strain in genetic crosses	this work
HZS.495	*hmbBΔ::riboB*$^+$ *riboB2*	studies of sexual structure, RT-qPCR	this work
HZS.499	*hmbBΔ::riboB*$^+$ *riboB2 pabaA1*	parental strain in genetic crosses	this work (obtained by cross of HZS.212 with HZS.450)
HZS.531	*hmbCΔ::pabaA*$^+$ *pabaA1 riboB2*	parental strain in genetic crosses, studies of sexual structure, RT-qPCR	this work (obtained by cross of HZS.338 with HZS.451)
HZS.521	*hmbAΔ::riboB*$^+$ *riboB2 pabaA1*	studies of sexual structure, RT-qPCR	this work (obtained by cross of HZS.205 with HZS.450)
HZS.653	*hmbBΔ::riboB*$^+$ *riboB2 pantoB100, yA2*	recipient strain in transformation experiment	this work (obtained by cross of HZS.499 with HZS.324)
HZS.658	*hmbCΔ::pabaA*$^+$ *pabaA1 pantoB100*	recipient strain in transformation experiment	this work (obtained by cross of HZS.531 with HZS.98)
HZS.678	*hmbAΔ:: riboB*$^+$ *riboB2, pantoB100 veA*$^+$ and 1 copy integration of pAN-HZS-9 containing *hmbA*$^+$	reconstituted *hmbAΔ* strain for studies of sexual structure	this work (obtained by transformation of pAN-HZS-9 into recipient strain HZS.655)
HZS.679	*hmbCΔ::pabaA*$^+$ *pabaA1 pantoB100 veA*$^+$ and 1 copy integration of pAN-HZS-10 containing *hmbC*$^+$	reconstituted *hmbCΔ* strain for studies of sexual structure	this work (obtained by transformation of pAN-HZS-10 into recipient strain HZS.658)
HZS.680	*hmbBΔ::riboB, riboB2, pantoB100, yA2, veA+* and 1 copy integration of pAN-HZS-11 containing *hmbB*$^+$	reconstituted *hmbBΔ* strain for studies of sexual structure	this work (obtained by transformation of pAN-HZS-11 into recipient strain HZS.653)

References

1. Yu JH, Hamari Z, Han KH, Seo JA, Reyes-Dominguez Y, et al. (2004) Double-joint PCR: a PCR-based molecular tool for gene manipulations in filamentous fungi. Fungal Genet Biol 41: 973-981.

2. Balazs A, Pocsi I, Hamari Z, Leiter E, Emri T, et al. (2010) AtfA bZIP-type transcription factor regulates oxidative and osmotic stress responses in *Aspergillus nidulans*. Mol Genet Genomics 283: 289-303.

3. Karacsony Z, Gacser A, Vagvolgyi C, Scazzocchio C, Hamari Z (2014) A dually located multi-HMG-box protein of *Aspergillus nidulans* has a crucial role in conidial and ascospore germination. Mol Microbiol 94: 383-402.
