# Supplementary material for: HMGB proteins are required for sexual development in Aspergillus nidulans
Source: PLoS One. 2019 Apr 25;14(4):e0216094. doi: 10.1371/journal.pone.0216094 (PMC6483251; doi:10.1371/journal.pone.0216094)
Supplement: S3 Table — (PDF) [file pone.0216094.s003.pdf]

S3 Table. Used primers

**Deletion of *hmbA* and *hmbC***	
hmbA up frw	5'- ctctgacctgccacgaggccttgctctatg-3'
hmbA up rev	5'- ggtgaaggtctgaagctgttggacgaagag3'
hmbA up nested frw	5'- ggagacatttcgaactgtatcagggctaac-3'
hmbA ribo chim frw	5'- *ctcttcgtccaacagcttcagaccttcacc*cgtacgtagtgtagattcaggcacattgaagcg -3'
hmbA ribo chim rev	5'-*cggaggtcaagcgactcgacactaaggtg*ggaaaactgccatgactactaggtggtgctatc-3'
hmbA down frw	5'- caccttagtgtcgagtcgcttgacctccg -3'
hmbA down rev	5'- gcatacaatgcgagcacggtggtcgtcatc -3'
hmbA down nested rev	5'- cggtgctgttgctgcttgaggacgaggag -3'
hmbC up frw	5'- gctgtattacgtcatgtacggagtag -3'
hmbC up rev	5- gcgaggaatgggtatgctagttg -3'
hmbC up nested frw	5'- ctgacattgagattctgagccacaac -3'
hmbC paba chim frw	5'- *caactagcatacccattcctcgc*gcacatagctattacacgtatgtttgagac -3'
hmbC paba chim rev	5'- *gaaaaccaatttactcgtcatcgcc*gtagtttgcttgaatggctaacgaggcattg -3'
hmbC down frw	5'- ggcgatgacgagtaaattggttttc -3'
hmbC down rev	5'- taatctgctcttcgatgccaattcc -3'
hmbC down nested rev	5'- catccgttgacgagccgatgatc -3'
hmbA down nested rev	5'- cggtgctgttgctgcttgaggacgaggag -3'
**PCR products for cloning**	
hmbA prom NotI frw	5'- tttttttgcggccgcgatcctcaatgaaccttgcccttg -3'
hmbA term NheI rev	5'- tttttttgctagcgtctgctgtgatattggaggacg -3'
hmbB NcoI frw	5'- tttttttccatggctctcaaactcattcgacg -3'
hmbB BamHI rev	5'- tttttttggatcccttcattcatccttgctcgatttcttgttc -3'
hmbC NcoI frw	5'- tttttttccatggctaaaaacgattcaaagaccg -3'
hmbC BamHI rev	5'- tttttttggatccttactcgtcatcgccaccgac -3'
**Other gene specific primers**	
hmbA frw	5'- atgcctaaggccaatcctacccgcaagacc -3'
hmbA rev	5'- ttaggacgactcctcatcctcttcggcttc -3'
hmbC frw	5'- cagccagctcaaatcgcttg -3'
hmbC rev	5'- cagcaaccttctcgcggtacg -3'
veA DM frw	5'- tgtgttatcccatcaagagg -3'
veA DM rev	5'- tctccgcgccgtctcatc -3'
veA frw	5'- agcccatccagcccatct -3'
veA rev	5'- tctccgcgccgtctcatc -3'
veA seq frw	5'- gacgacaagtatcgccttgaag -3'
**qPCR**	
hmbA ReTi frw	5'- aaagatgctcggtgagaagtg -3'

hmbA ReTi rev	5'- ctcgtaccgcttcttgtcag -3'
hmbB ReTi frw	5'- aagactacaaagaccaagaagacc -3'
hmbB ReTi rev	5'- ttgctcctcagtcagaacct -3'
hmbC ReTi frw	5'- cattcttcctgtacatgcatcac -3'
hmbC ReTi rev	5'- gtaccttcgtttgagacatcct -3'
actin ReTi frw2	5'- accatgtaccctggtatctc -3'
actin ReTi rev2	5'- ggaggagcaatgatcttgac -3'
**RT-qPCR**	
matB ReTi frw	5'- tcaactttctacccacttctcag -3'
matB ReTi rev	5'- atctccttctttcgcttgtcc -3'
matA ReTi frw	5'- gctatgaaatcaccaacacagtc -3'
matA ReTi rev	5'- ggacttcatcattcgtagaccc -3'
actA ReTi frw	5'- ggtatcatgatcggtatggg -3'
actA ReTi rev	5'- tatctgagtgtgaggatacca -3'
gpdA ReTi frw	5'- gacatcgtctctaccgacct -3'
gpdA ReTi rev	5'- gcttgatgaagttggagttgag -3'
eEF3 ReTi frw	5'- aactccttacaagccgtatcag -3'
eEF3 ReTi rev	5'- tcagaccgcaaacaatctcc -3'
tubC ReTi frw	5'- agacattctgcttggataacga -3'
tubC ReTi rev	5'- gagatcaccataagaaggcga -3'
hhtA ReTi frw	5'- gtaagtcctcactttgttgcc -3'
hhtA ReTi rev	5'- gtaaagttagcatcgtgaagcag -3'

Underlined letters in the primer sequences or in the primer names refer to the restriction sites designed within.
Italic letters at the 5' end refer to the chimeric nature of the primer.
