# Supplementary material for: HMGB proteins are required for sexual development in Aspergillus nidulans
Source: PLoS One. 2019 Apr 25;14(4):e0216094. doi: 10.1371/journal.pone.0216094 (PMC6483251; doi:10.1371/journal.pone.0216094)
Supplement: S1 Materials and methods — (PDF) [file pone.0216094.s004.pdf]

Supplementary Materials and methods

1. Construction of reconstitution vectors.

The reconstitution vectors for *hmbC* and *hmbB* were created by cloning the coding sequences from start codon to stop codon into the NcoI and BamHI sites of pAN-HZS-1 (see below the vector map with unique restriction sites shown). The NcoI / BamHI digestion of the pAN-HZS-1 vector results the excision of the GFP sequence. Therefore the reconstitution *hmbB* and *hmbC* vectors do not contain the GFP sequence.

The *hmbB* and *hmbC* genes are driven by the P_{gpdA} promoter.

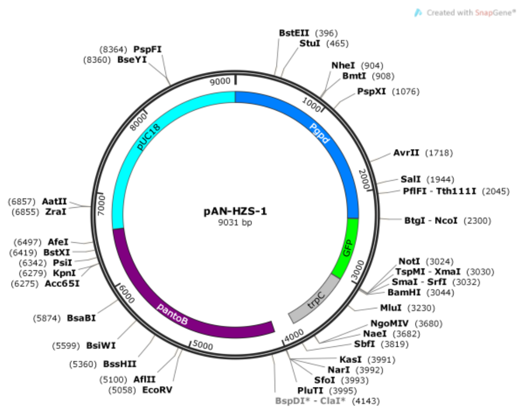

Figure: Structure of the pAN-HZS-1 vector with unique restriction sites shown.
pUC18: sequence of *E. coli* vector pUC18; Pgpd: promoter sequence of the *gpdA* gene of *A. nidulans*; GFP: coding sequence of green fluorescent protein coding gene; trpC: terminal sequence of the *trpC* gene of *A. nidulans*; pantoB: *pantoB* gene of *A. nidulans*.

The reconstitution vector for *hmbA* was created by cloning the *hmbA* PCR product amplified with "hmbA prom NotI frw" and "hmbA term NheI rev" primers into the NotI / NheI sites of the pAN-HZS-1 vector. This PCR product contains an 1843 nt long sequence upstream to the start codon of *hmbA,* the 489 nt long *hmbA* coding sequence from start codon to stop codon and a 918 nt long sequence downstream to the stop codon of *hmbA*. The region upstream to the start codon carries the promoter of *hmbA*, while the region downstream to the stop codon carries the terminal sequence of the *hmbA*. The PCR product was cloned into the NotI / NheI sites, therefore the orientation of the cloned gene relative to the P_{gpdA} promoter of the vector is opposite. This means that the *hmbA* gene in the reconstitution strain is driven by its native promoter.

The *hmbB* and *hmbC* reconstitution vectors were developed earlier than the *hmbA* reconstitution vector. We reasoned to change the operating promoter in case of *hmbA* in order to get an expression

level as close to the native state as possible, however, it is important to mention that it was integrated into an ectopic region, which might also has an effect on the expression level. In line with this potential *in trans* effect, we did not observe full complementation in the case of *hmbA* reconstitution.

We made full investigation of the time-course of *hmbA* and *hmbC* expression (will be published elsewhere) - similarly to that carried out for *hmbB* (Karacsony et al, 2014) – and found steady-state and high expression levels, thereby the usage of constitutive promoters for complementation studies sounds also reasonable in our case.

2. RT-qPCR

Reference genes

According to the MIQE guidelines (Bustin et al., 2009), we monitored the expression of five housekeeping genes in three biological replicates per mutant strain. These genes included *gpdA*, *eEF-3*, *tubC*, *hhtA* and actA. They encodes for Glyceraldehyde-3-phosphate dehydrogenase (*gpdA*/AN8041), Elongation factor 3 (*eEF-3*/AN6700), beta-tubulin (*tubC*/AN6838), histone H3 (*hhtA*/AN0733) and gamma-actin (*actA/AN6542*). The Cq values (see S1 Data) were analysed by the geNorm software (https://genorm.cmgg.be/) that calculates a gene expression stability measure (M) for the candidate reference genes (Vandesompele et al., 2002). According to this analysis, *gpdA* and *tubC* was found to be the most stable reference genes and thereby selected for the calculation of the expression normalization factor (see S1 Data). The expression normalization factor was calculated by taking the geometric mean of the expression levels (determined by standard curve analysis) of the selected *gpdA* and *tubC* reference genes. The expression normalization factor was calculated for each replicated datasets. The resulted normalization factors were then used to normalise gene expression values of genes of interest (*matA* and *matB,* Fig 5).

References

Bustin, S. A., et al., 2009. The MIQE guidelines: minimum information for publication of quantitative real-time PCR experiments. Clin Chem. 55**,** 611-22.

Vandesompele, J., et al., 2002. Accurate normalization of real-time quantitative RT-PCR data by geometric averaging of multiple internal control genes. Genome Biol. 3**,** RESEARCH0034.

RNA quality

We used agarose gel electrophoresis to assess the RNA quality (below).

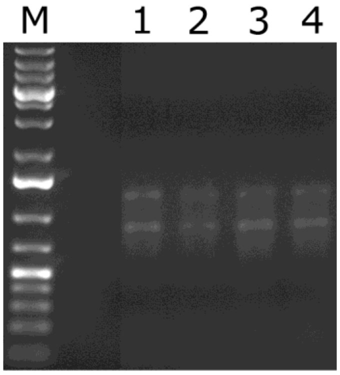

Figure: Gel electrophoresis of RNA samples derived from one of the three biological replicates.

Approx. 400 ng of RNAs were run on 1% TAE agarose gel with 8V/cm parameter. M: 1 kb DNA ladder Plus (Fermentas); Samples in lanes 1-4 are from the fourth day of sexual development (96 h after the induction of the sexual development). Lane 1: *veA*$^{+}$ control (HZS.450); lane 2: *hmbAΔ veA*$^{+}$ (HZS.521); Lane 3: *hmbBΔ veA*$^{+}$ (HZS.495); Lane 4: *hmbCΔ veA*$^{+}$ (HZS.531)

Genomic DNA contamination, DNase treatment

RNA samples were treated with RNase-Free DNase Set (Qiagen) according to the manufacturer's instructions. When RNeasy Plus Mini Kit (Qiagen) was used for RNA extraction, the DNase treatment with RNase-Free DNase Set (Qiagen) was conducted on the RNA binding columns. Here we must note that the extraction kit contained gDNA binding columns, which were extremely efficient and practically depleted the total DNA content of the RNA samples to zero. When TRIsure reagent (Bioline) was used for RNA extraction, the DNase treatment was done with RNase-Free DNase Set (Qiagen) in liquid according to the manufacturer's instructions. Usually DNase treatments following TRIsure reagent extraction were repeated two-three times in order to reach DNA-free quality.

DNA contamination of the RNA samples was checked by performing qPCR on 1 µg RNA samples with gamma-actin coding *actA* specific primers (actin ReTi frw2: 5’- accatgtaccctggtatctc -3’ and actin ReTi rev2: 5’- ggaggagcaatgatcttgac -3’) that do not span intron sequences. When Cq was lower than 32 cycle, the DNase treatment was repeated. Samples showing higher than 32 cycle Cq values in the DNA contamination test were used for reverse transcription.
