# Supplementary material for: HMGB proteins are required for sexual development in Aspergillus nidulans
Source: PLoS One. 2019 Apr 25;14(4):e0216094. doi: 10.1371/journal.pone.0216094 (PMC6483251; doi:10.1371/journal.pone.0216094)
Supplement: S1 Fig — Panel A. Schematic representation of the genomic region of hmbA+. The red and blue segments represent the sequence regions used to target the genomic regions by homologous recombination (HR1: homologous recombination sequence upstream to the deletion target, HR2: homologous recombination sequence downstream to the deletion target). The total DNAs of the hmbA+ control strain and putative deleted transformants were digested with XbaI restriction endonuclease. Zig-zag arrows show the positions of the XbaI cleavage sites. The Southern blot of XbaI digested total DNAs was probed with a digoxigenine labelled PCR product, as indicated in the scheme ("Probe"). Green and yellow boxes indicate the targeted hmbA gene and the riboB+ selection marker gene used for the gene-substitution, respectively. Arrows show the size of the hybridizing DNA fragments obtained by XbaI digestion. Panel B. Schematic representation of the substitution cassette constructed by the Double-Joint PCR method [40] (carrying the riboB+ selection marker gene) at the bottom of the panel and the arrangement of the targeted genomic region after the gene substitution event (by double cross overs between HR1 and HR2 regions) at the top of the panel. Dashed lines indicate homologous recombination events. Zig-zag arrows show the positions of the XbaI cleavage sites in the gene-substituted genomic region. Panel C. Image of the Southern hybridisation filter showing the hmbA+ signal on the left and the hmbAΔ signal on the right. The hmbA+ strain is the recipient parent HZS.120 and the presented deletion mutant is the HZS.205. (PDF) [file pone.0216094.s006.pdf]

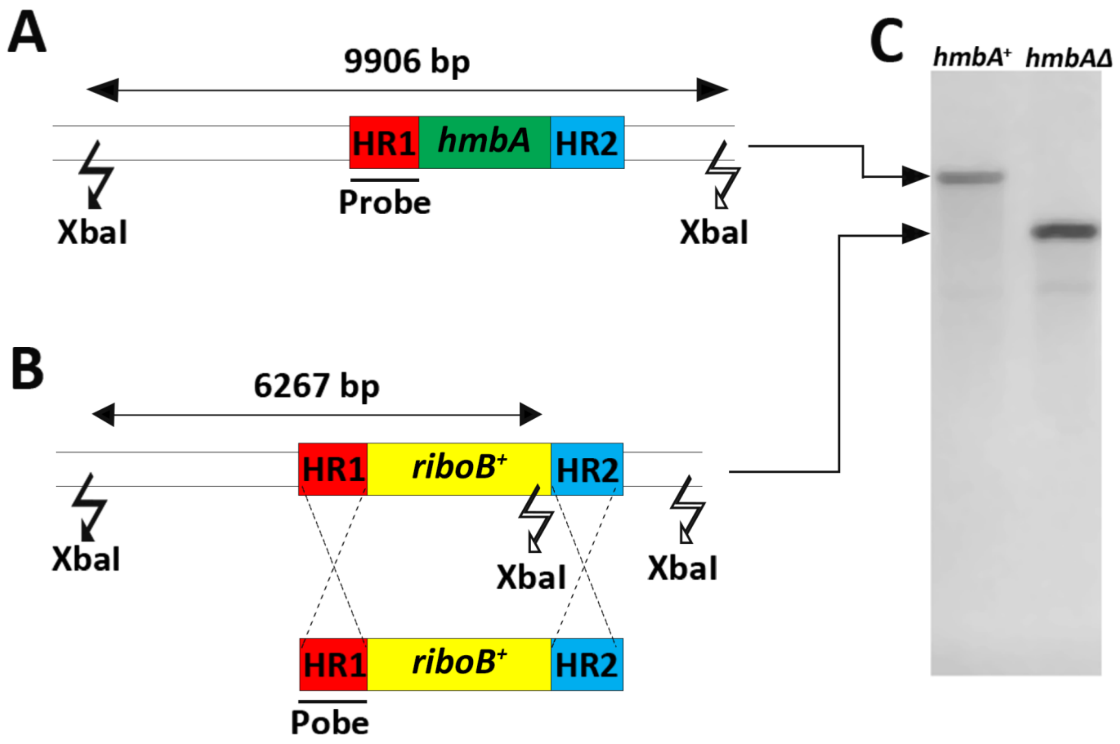

S1 Fig. Verification of single copy integration events in *hmbA* deletion transformants by Southern analysis. Panel A. Schematic representation of the genomic region of *hmbA*$^{+}$. The red and blue segments represent the sequence regions used to target the genomic regions by homologous recombination (HR1: homologous recombination sequence upstream to the deletion target, HR2: homologous recombination sequence downstream to the deletion target). The total DNAs of the *hmbA*$^{+}$ control strain and putative deleted transformants were digested with XbaI restriction endonuclease. Zig-zag arrows show the positions of the XbaI cleavage sites. The Southern blot of XbaI digested total DNAs was probed with a digoxigenine labelled PCR product, as indicated in the scheme ("Probe"). Green and yellow boxes indicate the targeted *hmbA* gene and the *riboB*$^{+}$ selection marker gene used for the gene-substitution, respectively. Arrows show the size of the hybridizing DNA fragments obtained by XbaI digestion.

Panel B. Schematic representation of the substitution cassette constructed by the Double-Joint PCR method [1] (carrying the *riboB*$^{+}$ selection marker gene) at the bottom of the panel and the arrangement of the targeted genomic region after the gene substitution event (by double cross overs between HR1 and HR2 regions) at the top of the panel. Dashed lines indicate homologous recombination events. Zig-zag arrows show the positions of the XbaI cleavage sites in the gene-substituted genomic region.

Panel C. Image of the Southern hybridisation filter showing the *hmbA*$^{+}$ signal on the left and the *hmbAΔ* signal on the right. The *hmbA*$^{+}$ strain is the recipient parent HZS.120 and the presented deletion mutant is the HZS.205.

References

1. Yu JH, Hamari Z, Han KH, Seo JA, Reyes-Dominguez Y, et al. (2004) Double-joint PCR: a PCR-based molecular tool for gene manipulations in filamentous fungi. Fungal Genet Biol 41: 973-981.
