# Supplementary material for: HMGB proteins are required for sexual development in Aspergillus nidulans
Source: PLoS One. 2019 Apr 25;14(4):e0216094. doi: 10.1371/journal.pone.0216094 (PMC6483251; doi:10.1371/journal.pone.0216094)
Supplement: S3 Fig — The strains were incubated on CM for 2 days at 37°C. Strains used: veA+ control (HZS.450), veA1 control (HZS.145), hmbAΔ veA+ (HZS.521), hmbAΔ veA1 (HZS.239), hmbBΔ veA+ (HZS.495), hmbBΔ veA1 (HZS.280), hmbCΔ veA+ (HZS.531), hmbCΔ veA1 (HZS.338), hmbAΔ veA+ hmbA reconstituted (HZS.678), hmbAΔ veA1 hmbA reconstituted (HZS.621), hmbBΔ veA+ hmbB reconstituted (HZS.680), hmbBΔ veA1 hmbB reconstituted (HZS.677), hmbCΔ veA+ hmbC reconstituted (HZS.679), hmbCΔ veA1 hmbC reconstituted (HZS.676). The complete genotypes are listed in S2 Table. (PDF) [file pone.0216094.s008.pdf]

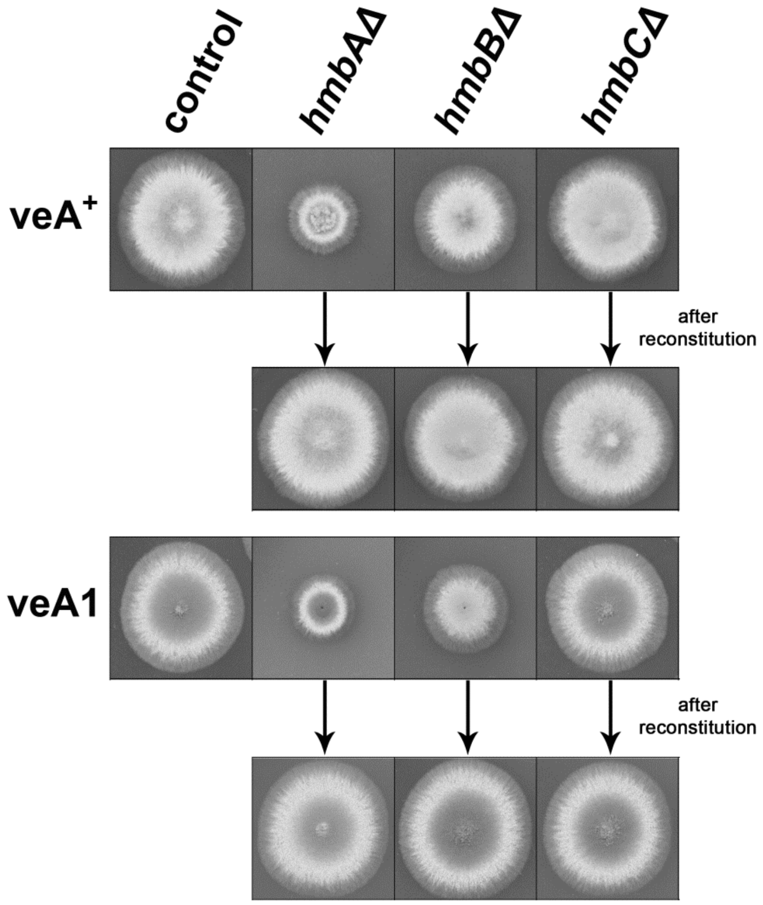


**S3 Fig. Growth ability of *veA⁺* and *veA1* controls, *hmbAΔ*, *hmbBΔ* and *hmbCΔ* strains and their cognate complementation (reconstitution) strains in both *veA⁺* and *veA1* background.** The strains were incubated on CM for 2 days at 37°C. Strains used: *veA⁺* control (HZS.450), *veA1* control (HZS.145), *hmbAΔ veA⁺* (HZS.521), *hmbAΔ veA1* (HZS.239), *hmbBΔ veA⁺* (HZS.495), *hmbBΔ veA1* (HZS.280), *hmbCΔ veA⁺* (HZS.531), *hmbCΔ veA1* (HZS.338), *hmbAΔ veA⁺ hmbA* reconstituted (HZS.678), *hmbAΔ veA1 hmbA* reconstituted (HZS.621), *hmbBΔ veA⁺ hmbB* reconstituted (HZS.680), *hmbBΔ veA1 hmbB* reconstituted (HZS.677), *hmbCΔ veA⁺ hmbC* reconstituted (HZS.679), *hmbCΔ veA1 hmbC* reconstituted (HZS.676). The complete genotypes are listed in S1 Table.
