# Supplementary material for: HMGB proteins are required for sexual development in Aspergillus nidulans
Source: PLoS One. 2019 Apr 25;14(4):e0216094. doi: 10.1371/journal.pone.0216094 (PMC6483251; doi:10.1371/journal.pone.0216094)
Supplement: S4 Fig — Cleistothecia were collected from colonies, and subsequently purified by rolling them on a sterile agar plate and documented by a camera in the presence of a ruler. Scale bar on the figure shows 1000 μm. Strains used: veA+ control (HZS.450), veA1 control (HZS.145), hmbAΔ veA+ (HZS.521), hmbAΔ veA1 (HZS.239), hmbBΔ veA+ (HZS.495), hmbBΔ veA1 (HZS.280), hmbCΔ veA+ (HZS.531), hmbCΔ veA1 (HZS.338), hmbAΔ veA+ hmbA reconstituted (HZS.678), hmbAΔ veA1 hmbA reconstituted (HZS.621), hmbBΔ veA+ hmbB reconstituted (HZS.680), hmbBΔ veA1 hmbB reconstituted (HZS.677), hmbCΔ veA+ hmbC reconstituted (HZS.679), hmbCΔ veA1 hmbC reconstituted (HZS.676). The complete genotypes are listed in S2 Table. (PDF) [file pone.0216094.s009.pdf]

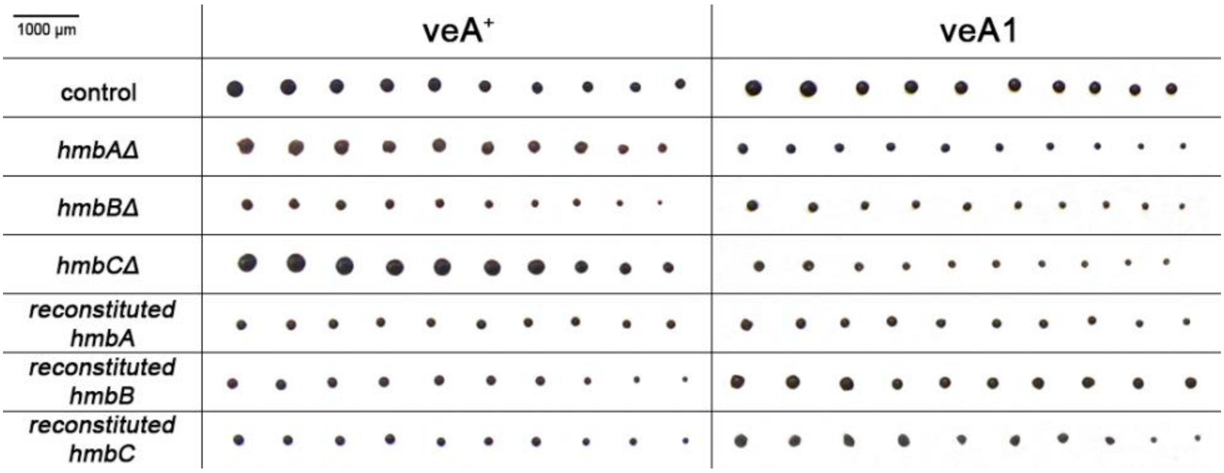

S4 Fig. Comparison of cleistothecia-sizes produced by *veA$^+$* and *veA1* controls, *hmbAΔ*, *hmbBΔ* and *hmbCΔ* strains and their cognate complementation (reconstituted) strains in both *veA$^+$* and *veA1* background. Cleistothecia were collected from colonies, and subsequently purified by rolling them on a sterile agar plate and documented by a camera in the presence of a ruler. Scale bar on the figure shows 1000 µm. Strains used: *veA$^+$* control (HZS.450), *veA1* control (HZS.145), *hmbAΔ veA$^+$* (HZS.521), *hmbAΔ veA1* (HZS.239), *hmbBΔ veA$^+$* (HZS.495), *hmbBΔ veA1* (HZS.280), *hmbCΔ veA$^+$* (HZS.531), *hmbCΔ veA1* (HZS.338), *hmbAΔ veA$^+$ hmbA* reconstituted (HZS.678), *hmbAΔ veA1 hmbA* reconstituted (HZS.621), *hmbBΔ veA$^+$ hmbB* reconstituted (HZS.680), *hmbBΔ veA1 hmbB* reconstituted (HZS.677), *hmbCΔ veA$^+$ hmbC* reconstituted (HZS.679), *hmbCΔ veA1 hmbC* reconstituted (HZS.676). The complete genotypes are listed in S1 Table.
