# Supplementary material for: HMGB proteins are required for sexual development in Aspergillus nidulans
Source: PLoS One. 2019 Apr 25;14(4):e0216094. doi: 10.1371/journal.pone.0216094 (PMC6483251; doi:10.1371/journal.pone.0216094)
Supplement: S5 Fig — Strain HZS.338 was incubated on CM for 4 days at 37°C. Sectoring areas are magnified. (PDF) [file pone.0216094.s010.pdf]

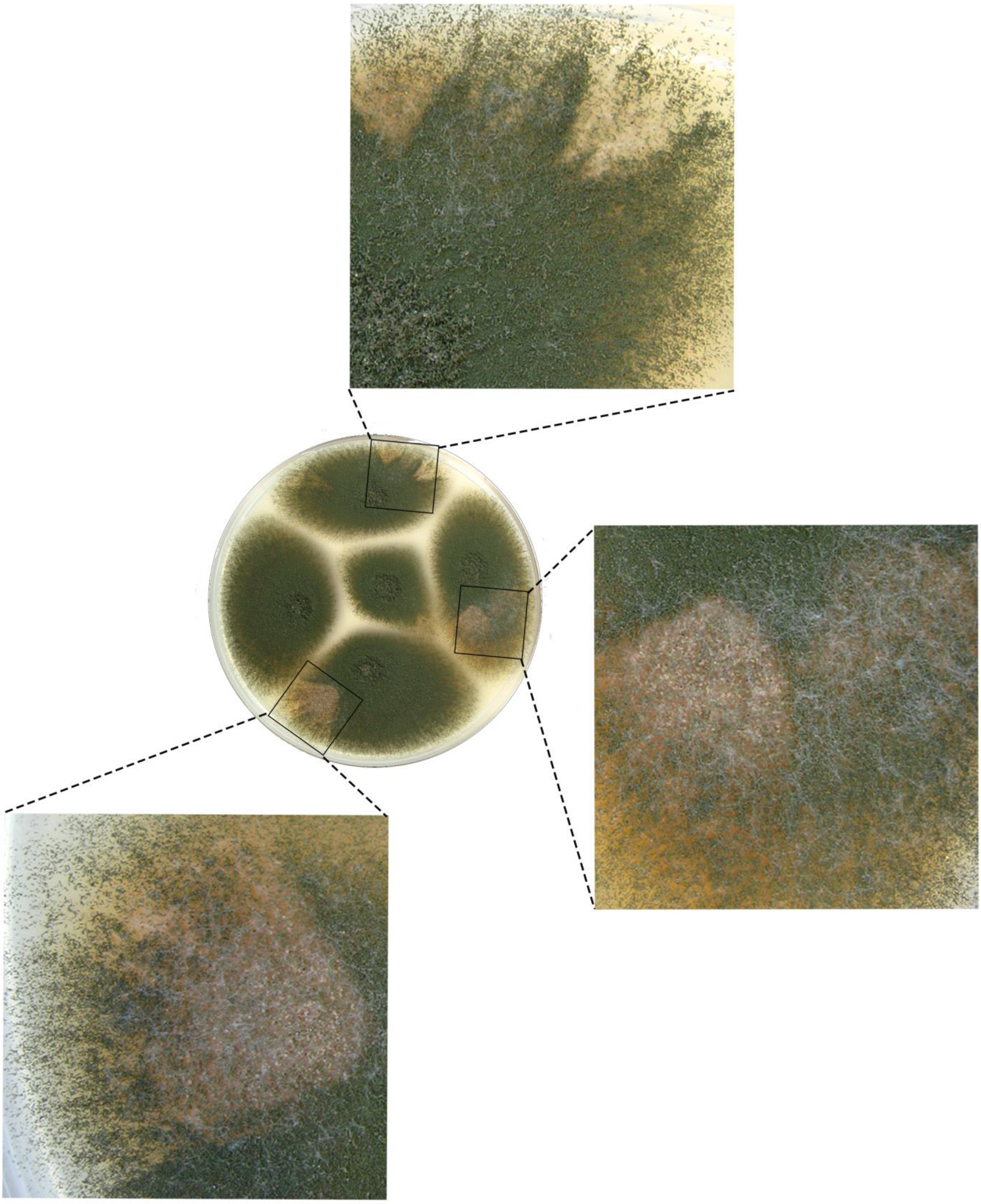

S5 Fig. Sectoring of *veA1 hmbCΔ* colonies. Strain HZS.338 was incubated on CM for 4 days at 37°C. Sectoring areas are magnified.
